# Global Patterns of Abundance, Diversity and Community Structure of the *Aminicenantes* (Candidate Phylum OP8)

**DOI:** 10.1371/journal.pone.0092139

**Published:** 2014-03-17

**Authors:** Ibrahim F. Farag, James P. Davis, Noha H. Youssef, Mostafa S. Elshahed

**Affiliations:** Department of Microbiology and Molecular Genetics, Oklahoma State University, Stillwater, Oklahoma, United States of America; Cairo University, Egypt

## Abstract

We investigated the global patterns of abundance, diversity, and community structure of members of the *Aminicenantes* (candidate phylum OP8). Our aim was to identify the putative ecological role(s) played by members of this poorly characterized bacterial lineages in various ecosystems. Analysis of near full-length 16S rRNA genes identified four classes and eight orders within the *Aminicenantes*. Within 3,134 datasets comprising ∼1.8 billion high throughput-generated partial 16S rRNA genes, 47,351 *Aminicenantes*-affiliated sequences were identified in 913 datasets. The *Aminicenantes* exhibited the highest relative abundance in hydrocarbon-impacted environments, followed by marine habitats (especially hydrothermal vents and coral-associated microbiome samples), and aquatic, non-marine habitats (especially in terrestrial springs and groundwater samples). While the overall abundance of the *Aminicenantes* was higher in low oxygen tension as well as non-saline and low salinity habitats, it was encountered in a wide range of oxygen tension, salinities, and temperatures. Analysis of the community structure of the *Aminicenantes* showed distinct patterns across various datasets that appear to be, mostly, driven by habitat variations rather than prevalent environmental parameters. We argue that the detection of the *Aminicenantes* across environmental extremes and the observed distinct community structure patterns reflect a high level of intraphylum metabolic diversity and adaptive capabilities that enable its survival and growth in a wide range of habitats and environmental conditions.

## Introduction

During the last quarter century, culture-independent diversity surveys have been extensively utilized to investigate bacterial diversity in almost all accessible habitats on earth [1]–[5]. These surveys have collectively demonstrated that the scope of bacterial diversity is much broader than previously expected based on culture-based assessments [6], [7], with a large fraction of the 16S rRNA gene sequences encountered not belonging to known cultured bacterial phyla. The term candidate phylum (CP) was thus proposed to describe such lineages [2].

One of the most important challenges facing microbial ecologists is to elucidate the putative metabolic capabilities and ecological roles of these candidate phyla, as well as the underlying ecological factors controlling their observed patterns of abundance, diversity, and community structure on a global scale. Various environmental genomics approaches have been utilized to obtain genomic fragments and partial genome assemblies from these lineages. These include construction and screening of large insert (Fosmid and BAC) libraries [8]–[11], direct metagenomic surveys and subsequent implementation of novel binning approaches to reconstruct genomes from metagenomic sequence data [12]–[15], and single cell genomics [16]–[19]. Collectively, these efforts have yielded valuable insight regarding the genomic characteristics and putative metabolic capabilities of multiple novel candidate phyla. Further, in several incidents, these insights were successfully utilized as a stepping-stone for enrichment and isolation of some of these lineages [20]–[22].

Genomic approaches are extremely valuable for deciphering putative metabolic capabilities of uncultured bacterial lineages. However, information from genomic studies is derived from a single sampling event in a single environment, and often from a single cell within the sample [17], [19]. Extrapolation of such information to imply similar capabilities and genomic features to all members and lineages within an entire bacterial phylum is hence inappropriate. This is especially true since a single bacterial phylum could exhibit a bewildering array of metabolic capabilities.

A complementary approach that has previously been utilized on an ecosystem level [23]–[27], but rarely utilized in a global phylocentric context, relies on using *in silico* database mining approaches to examine patterns of distribution of members of a specific candidate phyla in 16S rRNA gene diversity surveys. This approach could clarify the patterns of abundance, diversity, and community structure of the targeted lineage. This phylocentric strategy could greatly benefit from the dramatic increase in the number and size of publicly available 16S rRNA gene datasets; brought about by utilizing next generation sequencing technologies in recent ambitious initiatives to catalogue 16S rRNA gene diversity on a global scale [28]–[30].

Here, we describe a comprehensive examination of the global distribution of members of the *Aminicenantes* (candidate phylum OP8) using *in silico* database mining approaches. Our aim was to understand the putative ecological role(s) played by members of this poorly characterized bacterial lineages in various ecosystems and to demonstrate the utility of in silico database mining approaches in extracting meaningful ecological patterns from high throughput 16S rRNA gene datasets. Candidate phylum OP8 was first identified in sediments from the Obsidian Pool in Yellowstone National Park [2]. Since then, it has subsequently been identified in a wide range of terrestrial and marine habitats [31]–[34]. A recent study has described two near candidate phylum OP8 genome assemblies from 38 partial single cell genomes obtained from deep sediments of a brackish lake (Sakinaw lake, British Columbia, Canada), and the name *Aminicenantes* was proposed for this candidate phylum to highlight the high proportion of genes encoding aminolytic enzymes identified in both assemblies [16]. Our results highlight the ubiquitous nature of the *Aminicenantes*, and identify various environmental conditions impacting its global abundance and distribution in various habitats. We argue that these observed patterns suggest that, collectively, members of the *Aminicenantes* exhibit a high level of intra-phylum metabolic and adaptive diversities, and are hence capable of survival, and growth in a wide range of environmental extremes.

## Materials and Methods

### 1. A taxonomic outline of the *Aminicenantes*

While the candidate phylum *Aminicenantes* (CD-OP8) is recognized in several curated taxonomic outlines e.g. Greengenes [35] and SILVA [36], only a fairly low number of *Aminicenantes* sequences are deposited in these databases (109, and 12, in Greengenes and SILVA, respectively). The continuous deposition of new near full-length 16S rRNA gene sequences in GenBank database repository, coupled to the sporadic updates of curated taxonomic schemes, raises the prospect that additional *Aminicenantes* 16S rRNA sequences putatively representing novel high rank (class/ order) lineages have been deposited in GenBank but have yet to be included in taxonomic schemes. Therefore, as a preliminary step, we aimed to identify and classify all GenBank-deposited *Aminicenantes* 16S rRNA gene sequences and produce an updated and comprehensive taxonomic outline of this phylum. To this end, we queried GenBank NR database using BlastN [37], to identify the closest relatives of each of the 109 *Aminicenantes* sequences currently recognized in Greengenes and SILVA databases. The 500 closest relatives of each sequence were downloaded; and duplicates, sequences shorter than 800 bp, and chimeric sequences, identified using Galaxy [38], were removed. The remaining sequences (n = 2955) were aligned to a collection of reference sequences representing all *Aminicenantes* sequences, as well as sequences from a collection of 17 phyla, and 8 candidate phyla using ClustalX [39]. The phylogenetic positions of putative *Aminicenantes* sequences were evaluated using Distance, Parsimony, Maximum likelihood, and Bayesian approaches as previously described [40]. Sequences were deemed representative of a new class/order within the *Aminicenantes* if two or more distinct sequences remained reproducibly monophyletic and formed a bootstrap-supported independent clade upon varying the composition and size of the data set used for phylogenetic analysis [41].

### 2. Identification of *Aminicenantes* members in next Generation 16S rRNA gene datasets

Publicly available datasets generated using high throughput sequencing technologies (Pyrosequencing and Illumina) were downloaded from MG-RAST [42], VAMPS (http://vamps.mbl.edu/index.php), and GenBank SRA[43] (through the mirror web interface of DNA Databank of Japan http://www.ddbj.nig.ac.jp) in December 2012. Preliminary analysis indicated the absence of the *Aminicenantes* in human and metazoan microbiome samples and hence these datasets were excluded from further analysis (with the notable exception of rumen samples which were included). In total, 3,141 datasets from 110 different studies with 1,820,857,401 distinct 16S rRNA sequences were included in the analysis (Table S1 in File S1). All datasets were quality screened to filter all the sequences with lengths less than 50 base pairs, sequences with ambiguous nucleotides, and sequences with hompolymer stretches more than 8 bps. Sequences were classified using classify.seqs commands package in MOTHUR v.1.29.0 [44], using Silva alignment and Greengenes classification scheme. Sequences were identified as members of the *Aminicenantes* using a cutoff of 70% confidence threshold, as well as by confirmation of such assignment by sporadic manual insertion of putative *Aminicenantes* sequences into reference phylogenetic trees as described above. The subphylum level affiliation of all high throughput *Aminicenantes* sequences identified were determined using the updated taxonomic scheme produced in this study using near full length 16S rRNA gene sequences as described above. All analyses were conducted on a the HPC Cowboy super computer, a 252 compute nodes with dual six core CPUs and 32 GB RAMs server, 2 fat nodes with 256 GB RAM, GPU cards and 120 TB very fast disk storage at the OSU High Performance Computing Center at Oklahoma State University.

### 3. Classification of next-generation datasets according to habitat type and prevalent environmental conditions

All datasets included in this study were classified according to two different classification schemes: habitat type as well as prevalent environmental conditions. These classifications were used to determine the ecological prevalence and distribution patterns of various members of the *Aminicenantes*. Habitat-based classification scheme involved binning all 3,141 datasets into five major habitat types: Marine, aquatic non-marine, soil, hydrocarbon-impacted, and rumen/other (dust, animal-associated habitats and air). Due to the heterogeneity of geochemical and environmental conditions observed in marine, aquatic non-marine, and soil habitats, these three habitats were further sub-classified into multiple sub-habitat types, determined through the analysis of the projects’ available metadata (Table 1). For classification of datasets according to prevalent environmental conditions, three different classification schemes using temperature, oxygen tension**,** and salinity were utilized (Table 2). Classification based on prevalent pH conditions was not feasible due to the frequent absence of accurate pH metadata in a large proportion of the datasets, as well as the exceedingly low number of datasets that appear to originate from environments with preeminently low (e.g. <3) or high (e.g. >9) pH. A detailed description of all habitats examined in this study is presented in Table S1 in File S1.

**Table 1:** Classification and overall patterns of *Aminicenantes* relative abundance in various habitats and sub-habitats^1^.

Dataset type	Total datasets	Datasets with *Aminicenantes* (%)	Average *Aminicenantes* abundance (%)	Maximum relative abundance
Total datasets	3,141	918 (29.22%)	0.20%^2^	10.20%
Total 16S rRNA sequences	1,820,857,401	47,315	0.0026%	
Marine datasets	1,154	248 (21.50%)	0.28%	5.28%
Deep marine sediments	32	30	0.50%	2.89%
Coral associated microbiome	19	10	0.89%	4.67%
Pelagic	390	40	0.20%	2.46%
Hydrothermal vents	101	60	0.23%	5.28%
Coastal	612	107	0.20%	1.87%
Aquatic non-marine datasets	1,665	645 (38.74%)	0.15%	10.20%
Spring and ground water	25	10	2.80%	10.20%
Temperate freshwater	1569	587	0.11%	2.50%
Salt marshes	71	48	0.03%	0.67%
Soil datasets	276	14 (5.072%)	0.07%	0.80%
Agriculture	28	2	0.03%	0.06%
Grassland	140	10	0.00%	0.00%
Heavy metal/hydrocarbon contaminated	8	1	0.00%	0.01%
Arid and Semi-arid	46	0	0%	0%
Permafrost	54	1	0.01%	0.01%
Hydrocarbon-impacted datasets	14	10 (71.43%)	0.32%	0.95%
Herbivorous gut and other datasets^3^	32	1 (3.125%)	0.02%	0.02%

1 A detailed description of every dataset is provided as supplementary material (Table S1 in File S1).

2 Average abundance values in datasets where *Aminicenantes* sequences were identified.

3 26 Datasets were designated “other”; these datasets originated from dust, air and animal associated habitat. See Supplementary Table S1 in File S1 for details.

**Table 2:** Patterns of *Aminicenantes* relative abundance in datasets classified by prevalent environmental conditions^1^.

Dataset type	Total datasets	Datasets with *Aminicenantes* (%)	Average *Aminicenantes* abundance (%)^2^	Maximum relative abundance
Oxygen Tension				
Oxic	2,787	735(26.4%)	0.10%	2.50%
Hypoxic	101	35 (34.65%)	0.17%	2.90%
Anoxic	253	148 (58.5%)	0.46%	10.20%
Temperature^3^				
Low	317	4 (1.26%)	0.004%	0.01%
Temperate	2657	807 (30.372%)	0.19%	10.20%
Medium	53	48 (90.56%)	0.02%	0.06%
Elevated	11	6 (54.55%)	0.06%	0.20%
Extremely elevated	103	53 (51.46%)	0.24%	5.28%
Salinity^4^				
Non-Saline	1,863	575 (30.86%)	0.16%	10.20%
Low Salinity	1,179	274 (23.24%)	0.26%	5.30%
Moderate salinity	77	51 (66.23%)	0.02%	0.20%
Hypersaline	22	18 (81.81%)	0.07%	0.68%

1 A detailed description of every dataset is provided as supplementary material (Supplementary Table S1 in File S1).

2 Average abundance values in datasets where *Aminicenantes* sequences were identified.

3 Temperature classifications: Low: Arctic, Antarctic, subarctic, and permafrost marine and terrestrial conducive to the growth of psychrophilic microorganisms; temperate: Habitats in temperate ecosystems e.g. lakes, soils in continental settings; Medium: Habitats with temperatures around 37°C e.g. rumen; Elevated: habitats with temperatures conducive to the growth of thermophiles (50–80°C) e.g. Alberta oil sands tailings pond; Extremely elevated: habitats conducive to the growth of hyperthermophiles (>80°C degrees) e.g. Hydrothermal vents.

4 Salinity classifications: Non-saline: Environments with <1% salinity; Low salinity: Marine environments, and environments with comparable salinities; Moderate salinities: Environments with salinities around 5–15% e.g. Alberta oil sands tailings pond and Huabei Oilfield in China; Hypersaline: Environments with >15% salinity.

### 4. Deciphering ecological preferences and patterns of distribution of the *Aminicenantes*

The distribution and preferences of *Aminicenantes* were identified by correlating *Aminicenantes* relative abundance (% of sequences affiliated with *Aminicenantes* in the dataset), diversity, and community structure to its distribution in various habitats and sub-habitats, as well as across various environmental conditions.

Rarefaction curves were used to compare diversities of *Aminicenantes* community in different datasets as previously described [45]. We chose rarefaction curve analysis since it provides a sample size unbiased estimate of diversity and is hence useful in comparing datasets with wide variations in the numbers of sequences examined. In brief, rarefaction curves were constructed for all datasets with more than 50 sequences belonging to the *Aminicenantes*. Rarefaction curve plots were used to rank the datasets in order of diversity. Datasets with intersecting rarefaction curves were given the same rank. The datasets were ranked from one (least diverse) to 198 (most diverse) and subsequently binned into diversity categories as follows; “very low” (ranks 1–40), “low” (41–80), “medium” (81–120), “high” (121–160), and “very high” (161–198) categories. The ranks were then used to correlate *Aminicenantes* diversity to specific environmental factors using Spearman rank correlation and the significance of these correlations were tested in R [46].

The community structure profiles i.e. the proportion of various *Aminicenantes* lineages in various datasets were examined to reveal overall patterns of community structure in different habitats and under different environmental conditions. In addition, to zoom in on the patterns of *Aminicenante*s community structure in datasets where *Aminicenantes* represents a significant fraction of the overall bacterial community, the community structures in datasets with more than 50 *Aminicenantes* sequences (n = 198) were compared using principal-component analysis (PCA) and biplots were constructed using the R statistical package. In this analysis, the relative position of datasets is indicative of the level of their similarity, the directions of the class/subclass arrows are indicative of their respective maximal abundances, and the lengths of the arrows are proportional to the differential abundances of such lineages.

## Results

### 1. A revised taxonomic outline of the *Aminicenantes*

**Figure 1:**
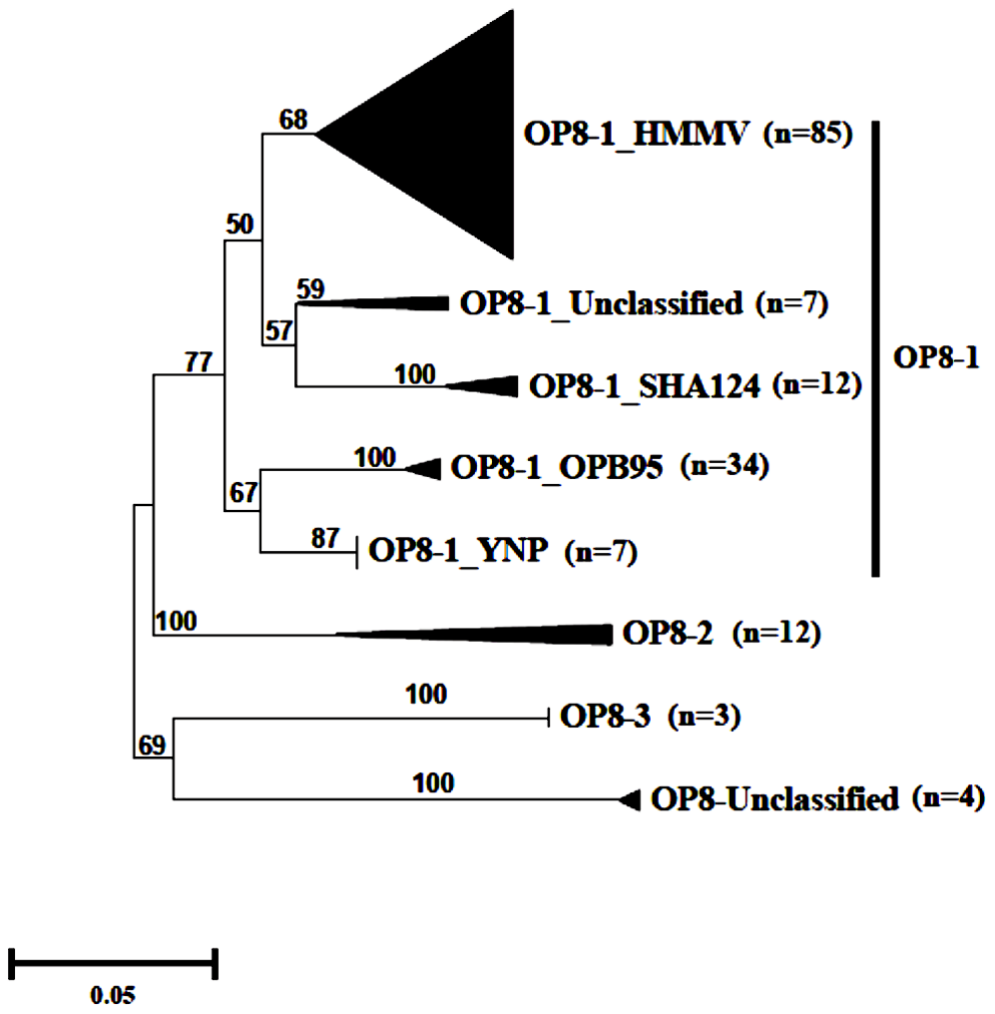
An updated taxonomic outline of the *Aminicenantes*. The Distance NJ tree was constructed using Jukes-Cantor corrections in MEGA5 [64]. Bootstrap values (in percent) are based on 1000 replicates and are shown for branches with more than 50% bootstrap support. Numbers in parentheses represent the number of sequences in each OP8 sub-phylum.

A total of 142 near-full length 16S rRNA *Aminicenantes* gene sequences were identified in GenBank NR database (Table S2 in File S1). Detailed phylogenetic analysis grouped the *Aminicenantes* sequences into four candidate classes: OP8-1, OP8-2, OP8-3 and OP8-unclassified. Candidate class OP8-1 has the largest number of near full-length *Aminicenantes* sequences and is comprised of five distinct orders (OP8-1_HMMV, OP8-1_SHA-124, OP8-1_OPB95, OP8-1_unclassified, and OP8-1_YNP) (Figure 1, Table S2 in File S1). In contrast, classes OP8-2, OP8-3, and OP8-unclassified have a lower number of near full-length sequences and are not further sub-classified into candidate orders. This revision of *Aminicenantes* phylogeny hence increased the number of recognized near full-length 16S rRNA gene sequences by 30.3%, and added one candidate class (OP8-3) and one candidate order (OP8-1_YNP) to the Greengenes taxonomic outline, the most detailed *Aminicenantes* classification scheme in curated databases.

### 2. Identification of members of *Aminicenantes* in next generation 16S rRNA gene datasets

We used pyrosequencing- and Illumina-generated 16S rRNA gene datasets available in three publicly available gene repositories (VAMPS, GenBank, and MG-RAST) [42], [43] to identify the patterns of relative abundance, diversity, and community structure of members of the *Aminicenantes*. Within 3,141 datasets comprising ∼1.8 billion 16S rRNA gene sequences, 47,315 (0.0026%) from 918 (29.2%) different datasets were affiliated with the *Aminicenantes* (Table 1, Table S1 in File S1).

### 3. Patterns of *Aminicenantes* abundance

Overall relative abundance of *Aminicenantes* varied widely between various datasets, and ranged between 0 and 10.2% (encountered in MG-RAST dataset number 4455892, obtained from groundwater heavily contaminated by arsenic in the Ganges-Brahmaputra Delta region of Bangladesh, [47] (Table 1, Figure S1 in File S1). Although *Aminicenantes* has been identified in a substantial fraction (29.2%) of examined datasets, it invariantly constituted a minor fraction of the bacterial community identified, and rarely exceeded 5% in all datasets (Table 1, Figure S1 in File S1).

Based on incidence of occurrence (i.e. percentages of datasets in which sequences affiliated with the *Aminicenantes* were identified), and relative abundance of *Aminicenantes* in various datasets (Table 1, Figure S1 in File S1), members of the *Aminicenantes* appear to be most abundant in hydrocarbon-impacted habitats, being identified in 71.4% of the datasets (10/14), with an average abundance of 0.321%. The *Aminicenantes* was also frequently identified in marine (21.5% of datasets) and aquatic non-marine (38.74% of datasets) habitats, with average relative abundances of 0.275%, 0.146%, respectively (Table 1, Figure S1 in File S1). On the other hand, the *Aminicenantes* were rarely identified in soils and rumen habitats (Table 1, Figure S1 in File S1).

*Aminicenantes* abundance also demonstrated distinct patterns in relation to oxygen tension, temperature, and salinity (Table 2, Figure S2 in File S1). The *Aminicenantes* were most abundant in anaerobic habitats (58% of datasets, average 0.46%) e.g. Mai Po mangrove marshes in Hong Kong, heavy metal contaminated ground water in Bangladesh [47], active hydrothermal vent sediments from the Mid-Atlantic Ridge [48], anoxic sulfide and sulfur-rich terrestrial spring in southwestern Oklahoma (Zodletone spring) [40], and anoxic sediments from the Guaymas [3] and Cariaco Basins [49] (Table S1 in File S1). However, the *Aminicenantes* were also identified in much lower abundance in few oxic habitats e.g. water and sediments from coastal and open ocean sites surveyed from South Atlantic to the Caribbean seabed (Table S1 in File S1), coastal water of western English channel [50], and soils and sediments of hypersaline lake, La Sal del Rey’s in southern Texas, USA [51] (Table S1 in File S1). Temperature profile of *Aminicenantes* abundance indicated an extremely rare occurrence in low temperature terrestrial and marine habitats (e.g. in datasets from the Canadian, Alaskan and European tundra and arctic soils, as well as the Amundsen sea [50], [52]), (Table S1 in File S1), and a slightly higher preference (based on incidence of occurrence) to habitats with temperate, medium, elevated, and extremely elevated temperatures (Table 2, Figure S2 in File S1). Salinity wise, *Aminicenantes* was present at all levels of salinities, with slightly higher relative abundances in non-saline, and low salinity habitats (Table 2, Figure S2 in File S1).

### 4. Patterns of *Aminicenantes* community structure

**Figure 2:**
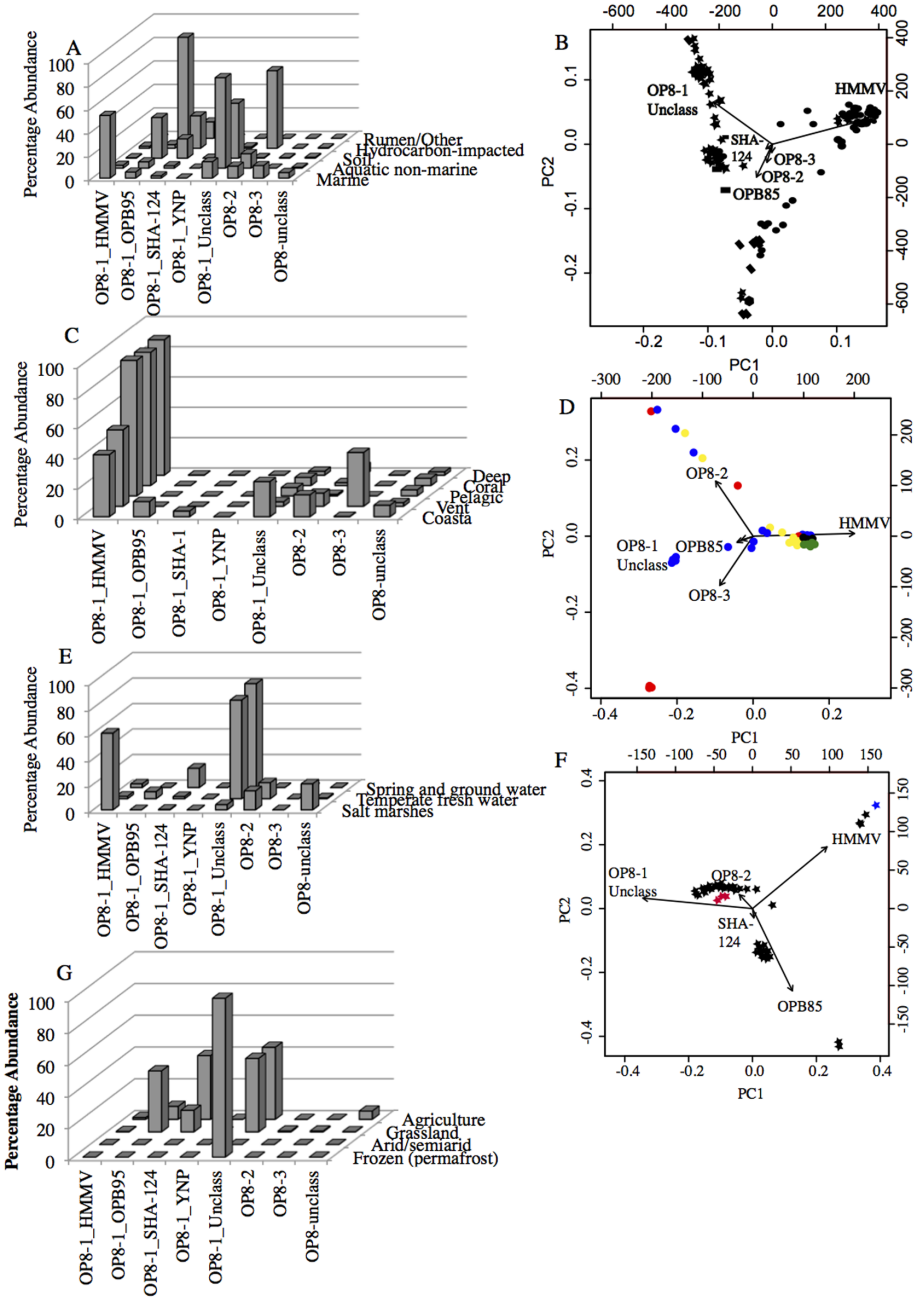
Aminicenantes relative abundance and community in various habitats. (A) Relative abundance of Aminicenantes-affiliated sequences in marine, aquatic non-marine, soil, hydrocarbon-impacted, and rumen/other habitats. (B) PCA biplot of the community structure of Aminicenantes in datasets belonging to marine (•), aquatic non-marine (★), soil (n), hydrocarbon-impacted (u), and rumen (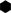) with >50 Aminicenantes sequences. The biplot was generated in R using the prcomp and biplot functions in library labdsv. The first 2 axes explained 73% of the variance. There are two sets of axis scales on the biplot; the ones on the right and top correspond to the axis scores for samples, and the bottom and left axes correspond to the loadings of the variables (in this case, OP8 subphyla). (C) Relative abundance of *Aminicenantes*-affiliated sequences in various marine subhabitats. (D) PCA biplot of the community structure of *Aminicenantes* in marine datasets classified as coastal (blue), pelagic (green), hydrothermal vent (red), coral-associated (black), and deep sediment (yellow). There are two sets of axis scales on the biplot; the ones on the right and top correspond to the axis scores for samples, and the bottom and left axes correspond to the loadings of the variables (in this case, OP8 subphyla). (E) Relative abundance of *Aminicenantes*-affiliated sequences in environments originating from aquatic non-marine habitats. (F) PCA biplot of the community structure of *Aminicenantes* in aquatic non-marine datasets classified as freshwater (black), spring and groundwater (red), and salt marshes (blue). There are two sets of axis scales on the biplot; the ones on the right and top correspond to the axis scores for samples, and the bottom and left axes correspond to the loadings of the variables (in this case, OP8 subphyla). (G) Relative abundance of *Aminicenantes*-affiliated sequences in environments originating from soil habitats. Since only one soil dataset contained >50 *Aminicenantes* sequence, a PCA soil biplot is not feasible.

Examination of patterns of *Aminicenantes* community composition across habitats revealed several distinct patterns. For example, order OP8-1_HMMV appears to be prevalent in marine environments, where it represented 53.5% of the total *Aminicenantes* sequences identified in marine datasets (Figure 2a). Class OP8-1_unclassified appeared to be the prominent lineage in aquatic non-marine environments, where it represented 77% of the total number of *Aminicenantes* sequences (Figure 2a). Order OP8-2 was the prevalent *Aminicenantes* lineage in hydrocarbon-impacted environments where it represented 66% of the total number of sequences. Although extremely rare in the rumen, the *Aminicenantes* sequences identified in a single dataset from this habitat belonged to order OP8-1_OPB95. PCA analysis conducted on datasets with more than 50 *Aminicenantes* sequences (n = 198, Figure 2b) confirmed such patterns where most of the environments from marine origins clustered along the OP8-1_HMMV species arrow (circles in Figure 2b), most of the environments from aquatic non-marine origins clustered along the OP8-1_unclassified species arrow (stars in Figure 2b), and the majority of the hydrocarbon-impacted environments clustered in the direction of the OP8-2 species arrow (diamonds in Figure 2b).

Sub-classification of habitats (Figures 2c-h) further revealed additional patterns at the sub-habitat level, especially in marine, soil, and aquatic non-marine habitats systems. Within marine environments, the prevalence of OP8-1_HMMV was more pronounced in coral-associated, pelagic, and deep marine datasets (Figure 2c). Indeed, in marine datasets with >50 *Aminicenantes* sequences, OP8-1_HMMV represents the majority (more than 80%) of the total *Aminicenantes* sequences in all coral-associated and pelagic datasets, as well as in the majority (10 out of 13) of deep sediment datasets. OP8-1_HMMV also represented the majority of *Aminicenantes* sequences in a few of the coastal (three out of 15) and hydrothermal (one out of six) datasets. Accordingly, those samples clustered together along the OP8-1_HMMV species arrow in the PCA biplot (red circles representing one vent sample, green circles representing five pelagic samples, yellow circles representing ten deep sediment samples, black circles representing five coral-associated samples, and blue circles representing three coastal samples in Figure 2d). In the remaining marine samples, the majority of *Aminicenantes* datasets has a mixed community of OP8-1_HMMV and other lineages, and so had an intermediary position between species arrows in the PCA biplot. In rare cases, some datasets did not contain any OP8-1_HMMV sequences. For example, all *Aminicenantes*-affiliated sequences from three hydrothermal vent samples belonged to the newly proposed candidate class OP8-3, and hence clustered in the direction of OP8-3 species arrow in the PCA biplot (red circles in Figure 2d).

Within aquatic non-marine habitats, the overall majority of *Aminicenantes* sequences belonged to subclass OP8-1_unclassified (Figure 2e). The majority (85.1% of datasets originating from the two non-saline aquatic non-marine sub-habitats (temperate freshwater lakes, and spring and groundwater samples) showed >70% of *Aminicenantes*-affiliated sequences belonging to the order OP8-1_unclassified and were hence clustered along the OP8-1_unclassified arrow in the PCA biplot (black and red stars, Figure 2f). However, two notable exceptions to this pattern were observed: 1. In several datasets, a mixed community of OP8-1_ unclassified with other lineages was observed (e.g. 11 freshwater lake samples had a mixed community of OP8-1_unclassified (53.6±2.4%), OP8-1_OPB95 (34.2±3.2%), and OP8-1_SHA-124 (11.3±1.9%), and one sample from a sinkhole had a mixed *Aminicenantes* community of OP8-1_OPB95 (43.7%), OP8-1_unclassified (32.1%), and OP8-2 (22.4%). 2. In few datasets, OP8-1_unclassified order was absent e.g. sewage samples with high abundance (> 90%) of OP8-1_OPB95 (Figure 2f).

While the *Aminicenantes* class OP8-1_unclassified was the prevalent lineage in the majority of aquatic non-marine habitats originating from temperate freshwater lakes, as well as spring and groundwater datasets; a distinct community structure was observed in aquatic non-marine habitats with low to moderate salinity (Figure 2e). Within these habitats, e.g. three samples from the Amazon-Guianas estuaries, and a salt marsh samples from Cabo Rojo, PR, the majority of *Aminicenantes*-affiliated sequences belonged to order OP8-1_HMMV (83.7±7.98%). Accordingly, those samples clustered along the HMMV species arrow in various PCA plot (Figure 2f).

Finally, a relatively small number of *Aminicenantes*-affiliated sequences were present in soil samples. Those were mainly affiliated with orders OP8-1_unclassified, OP8-1_OPB95, and OP8-1_SHA-124. Some unique patterns were observed at the sub-habitat level e.g. the prevalence of OP8-1_unclassified order in samples from permafrost soils (Figure 2g). However, it is important to note that the *Aminicenantes* exhibited an extremely rare distribution in all soil datasets examined, being only identified in 14 out of 276 datasets, with an extremely low average relative abundance (0.07%). Therefore, the significance of the observed patterns, given their extreme rarity, and doubtful ecological role in soil habitats, is questionable.

**Figure 3:**
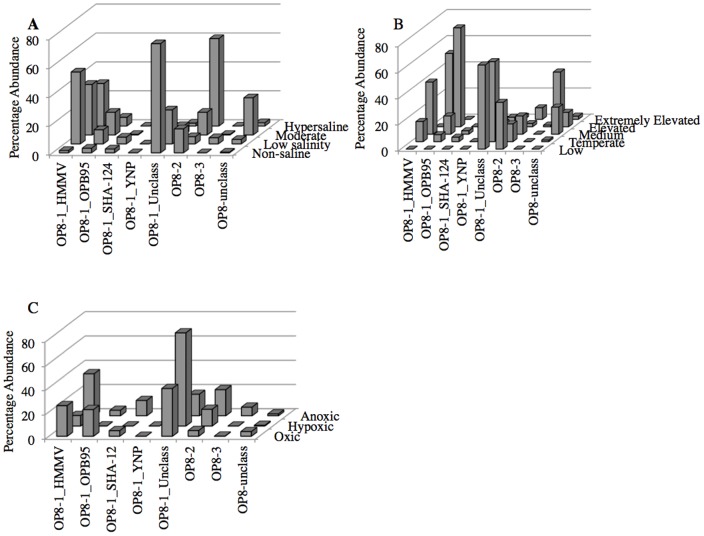
Relative abundance of *Aminicenantes*-affiliated sequences in different environments sub-classified according to (A) temperature, (B) oxygen tension, and (C) salinity.

We also studied the effect of environmental conditions (O_2_ tension, temperature, and salinity) on *Aminicenantes* community structure in various datasets. When environments were classified based on their salinity, we observed a shift in the prevalence of various *Aminicenantes* lineages, with order OP8-1_unclassified representing the majority of *Aminicenantes-*affiliated sequences in non-saline habitats, as opposed to order HMMV in low and moderate salinity environments, and class OP8-2 in hypersaline environments (Figure 3a). We also observed an effect of temperature on the pattern of *Aminicenantes* community structure changes, where order OP8-1_unclassified and class OP8_2 dominated in low temperature and psychrophilic habitats, as opposed to orders OP8-1_OPB95 and HMMV in thermophilic and hyperthermophilic habitats (Figure 3b). However, the uneven number of samples belonging to each category (Table 3) could possibly skew these results. Finally, no remarkable effect of O_2_ tension on *Aminicenantes* community structure was observed (Figure 3c).

**Table 3:** Diversity rankings of all datasets classified according to habitat and prevalent environmental conditions.

Habitat/ Environmental parameter	Average diversity rank±SD	Number of samples belonging to this diversity rank				
		Very low	Low	Medium	High	Very high
Marine	115±68.5	11	1	6	10	12
Pelagic	89.9±63.3	1	0	2	1	0
Coastal	169.1±39.6	0	1	0	1	7
Coral	115.4±57.3	1	0	1	3	0
Deep_sed	106.4±65.9	5	0	3	4	5
Hyd_vent	34.3±59.1	4	0	0	1	0
Non-marine	96±52.8	26	38	34	31	21
Freshwater	93.7±51.6	26	38	34	30	18
Spring/GW	187.5±8.8	0	0	0	0	3
Salt marsh	148	0	0	0	1	0
Hydrocarbon-Impacted Soil	96.5±83.4	3	0	0	0	4
Soil	174.5	0	0	0	0	1
Salinity						
Non-saline	95.8±52.4	29	38	34	29	24
Low-salinity	115.8±69.6	11	0	6	10	14
Hypersaline	115.3±47.4	0	1	0	2	0
Temperature						
Temperate	102.6±56.1	33	39	40	40	38
Elevated	24.3±47	7	0	0	1	0
O2 tension						
Anoxic	88.3±74.5	12	1	1	6	7
Hypoxic	84.6±44.1	26	36	37	27	5
Oxic	157.4±49.5	2	2	2	7	26

### 5. Patterns of *Aminicenantes* diversity

One hundred and ninety-eight datasets with more than 50 *Aminicenantes*-affiliated sequences were included in the diversity analysis. Due to the underrepresentation of hydrocarbon-impacted sites and soils, comparison of diversities was restricted to the marine and aqueous non-marine habitats and their subcategories. Within all habitats, the levels of diversity varied widely, but marine habitats showed higher diversity than freshwater habitats (Student t-test p-value = 0.037), with most of the marine environments (72%) showing medium to very high *Aminicenantes* diversity (Table 3). Within marine habitats, a higher average diversity rank was observed in coastal samples, and a lower average diversity was observed in hydrothermal vent samples. Indeed, coastal samples *Aminicenantes* diversities were significantly higher than those in all other marine environments (p-value ranging from 0.0004 to 0.041). Hydrothermal vent samples *Aminicenantes* diversities were significantly lower than those in coastal, and deep marine sediment samples (p-values 0.0004, and 0.04, respectively).

*Aminicenantes* diversity within aquatic non-marine environments varied, with high diversities observed in spring/groundwater samples and the single sample from a salt marsh. Significantly lower diversities were observed in samples from freshwater temperate environments (p-value = 0.002).

We also correlated diversity rankings to environmental conditions including temperature, salinity, and oxygen tension (Table 3). Interestingly, while no clear correlation was identified between temperature, or salinity and diversity levels of *Aminicenantes* at OTU_0.03_, a positive highly significant correlation existed between the dataset diversity rank and the environment’s oxygen tension (Spearman rank correlation coefficient = 0.4, p-value  = 5.3E-9).

## Discussion

In this study, we utilized *in silico* database mining approaches to provide an updated and expanded taxonomic outline of the candidate phylum *Aminicenantes* using near full-length 16S rRNA gene sequences, as well as to examine the global patterns of *Aminicenantes* distribution using high throughput (Pyrosequencing and Illumina) generated 16S rRNA gene datasets. We report that: 1. Members of the *Aminicenantes* are present in a substantial fraction (918 out of 3,141) of high throughput-generated datasets examined, where they represent a minor/rare fraction of the community, with very few exceptions. 2. Members of the *Aminicenantes* are ubiquitous, being encountered in all different types of habitats and across all spectra of environmental parameters (temperature, salinity, and oxygen tension) examined. 3. Distinct differences exist between the relative abundance of the *Aminicenantes* across different habitats and environmental conditions. 4. Members of the *Aminicenantes* exhibit a distinct community structure patterns across various datasets, and these patterns appear to be, mostly, driven by habitat variations rather than prevalent environmental parameters.

Utilizing high throughput-generated datasets of partial 16S rRNA gene sequences in dedicated sequence repositories (VAMPS, MG-RAST, and GenBank SRA) for analyzing patterns of prokaryotic diversity represents an extremely valuable, yet largely overlooked, resource. Next generation sequencing datasets are often deposited with a single accession number per dataset, often with inadequate metadata, and, unlike Sanger-generated sequences, these datasets are not readily amendable to online search queries. Nevertheless, when properly exploited, these datasets represent an excellent resource for testing specific ecological hypothesis. Examining *Aminicenantes* diversity in 3,141 distinct datasets, comprising a total of ∼1.8 billion partial sequences clearly demonstrates the presence of members of this candidate phylum in a large number (29.2% of datasets examined) of habitats. However, the *Aminicenantes* always represented a minor fraction of the overall community and often exhibited an extremely rare distribution: The relative abundance of the *Aminicenantes* was less than 0.01% of the total community in 70.1% of datasets examined, 0.01–0.1% in 16.1% of datasets examined, 0.1–1% in 12.9% of datasets, and more than 1% in only 0.9% of datasets examined (Table S1 in File S1). The reason for the occurrence, survival, and retention of various lineages as members of the rare biosphere (e.g. less than 0.1%) in various environments is an issue that has previously been thoroughly debated [6], [53], [54]. Possible reasons explaining this phenomenon vary and range between filling very specialized niches, acting as a backup system that readily responds to seasonal variations encountered in various ecosystem, exhibiting extremely slow growth or dormancy, introduction to the ecosystem through recent immigration of these rare phylotypes to the sampling site, or introduction to the dataset through contamination during sampling, DNA extraction, or amplification. Indeed, several of these explanations are plausible to elucidate the role of extremely rare members of the *Aminicenantes* in their respective ecosystems. Regardless, it is reasonable to assume that the detection of the *Aminicenantes* above a certain empirical threshold (e.g. 1%, equivalent to 10^5^ cells/gram or ml in a community with a cell count of 10^7^) reflects its successful colonization and propagation in a specific habitat, and suggests its importance in fulfilling vital ecosystem services that justifies its retention in that habitat. Therefore, examination of the few datasets in which the *Aminicenantes* are present in relatively higher abundances could offer a window on what factors are conducive for *Aminicenantes* survival and propagation *in-situ*. Datasets with more than 1% *Aminicenantes* relative abundance (Table S1, Figure S1 in File S1) (0.9% of the total number of datasets) were not restricted to one habitat type or one environmental condition, but occurred within the majority of the five habitats examined, and across a wide range of environmental conditions. Therefore, it is improbable that a single, specific, environmental condition e.g. hypersalinity or extreme temperature represents the only scenario for eliciting a competitive niche for the *Aminicenantes*. Rather, we argue that conditions at which *Aminicenantes* propagates appear to be induced by other types of natural or anthropogenic stressors, which effectively preclude a large fraction of the population, opening the window for *Aminicenantes* to propagate. This is apparent from the fact that many of the datasets with >1% *Aminicenantes* relative abundance came from environments with variable types of environmental stressors e.g. high levels of hydrocarbons (e.g. Alberta oil sands tailing ponds, Petroleum reservoirs in Huabei, China, and north slope oil facility) [55]–[57], or high levels of metal (arsenic) contamination in Araihazar, Bangladesh [47].

Overall relative abundance of the *Aminicenantes* appeared to vary widely across various habitats, as well as across specific environmental conditions. The *Aminicenantes* appear to be most abundant in hydrocarbon-impacted environments, being encountered in 71.4% of the datasets (10/14), with an average abundance of 0.321%. The association of specific lineages and phylotypes with hydrocarbon-impacted environments regardless of its origin (natural or anthropogenic), or chemical composition (natural gas, petroleum, enrichments on a single substrate) has previously been noted ([58], [59]. This prevalence in hydrocarbon-impacted settings is in agreement with the notion that success and propagation of members of the *Aminicenantes* in a specific environment is contingent on the occurrence of specific environmental stressors (hydrocarbon contamination and possibly associated anaerobiasis and high sulfide levels in such habitats) that partially alleviates competition, allowing for successful propagation of members of the *Aminicenantes*. The *Aminicenantes* were also identified in a considerable fraction of marine and aquatic non-marine habitats (Table 1, Table S1 in File S1). However, the complexity and variability of geochemical parameters encountered in these heterogeneous ecosystems prevents us from deciphering what exact environmental characteristics, or combination thereof, within these habitats favored *Aminicenantes* propagation. Correlating *Aminicenantes* abundance to environmental conditions (temperature, salinity, and oxygen tension) revealed that while members of the *Aminicenantes* could be encountered in a wide range of environmental conditions, it appears to exhibit significantly higher abundances in anoxic (compared to oxic and microoxic) habitats and a significantly lower abundance in low temperature (compared to temperate and elevated temperature) habitats. The relatively higher abundance of the *Aminicenantes* in anoxic environments suggests a prevalent anaerobic/facultative mode of metabolism within the *Aminicenantes*. Indeed, the majority of studies where the *Aminicenantes* represented more than 1% the total bacterial community originated from seemingly anaerobic habitats (e.g. arsenic contaminated ground water from Bangladesh, Guayamas methane seeps, and hypoliminion sites in Lake Mendota).

Analysis of the *Aminicenantes* community structure was conducted by: 1. Utilizing the classification of all (47,351) next generation sequences identified to examine the *Aminicenantes* community structure in various types of habitats and across various environmental conditions, and 2. PCA analysis of the *Aminicenantes* community structure in datasets where they exhibited relatively higher abundances (n>50). Overall, it appears that factors impacting *Aminicenantes* community structure are mostly habitat-driven (i.e. similar community structure observed in similar habitats), rather than driven by prevalent environmental conditions (temperature, salinity, oxygen tension) within an ecosystem. For example, class OP8-3 was exclusively identified in hydrothermal vent habitats; order OP8-1_ HMMV represented the majority of *Aminicenantes* sequences encountered in coral associated, pelagic, and deep marine habitats; OP8-1_unclassified represented the majority of sequences in aqueous non-marine habitats; and OP8-2 represented the majority of sequence in hydrocarbon-impacted habitats. The role of prevalent environmental condition in shaping the *Aminicenantes* microbial community is less certain, mostly due to the inadequate representation of special categories e.g. normal (body) temperature, elevated temperature, and hypersaline environments. However, one notable exception in which an environmental parameter appears to play a clear role in shaping the *Aminicenantes* microbial community is the distinct prevalence of order OP8-1_ HMMV in multiple low salinity datasets regardless of their habitat.

Finally, it is interesting to note that *Aminicenantes* sequences were identified across all ranges of salinity and temperatures including those conducive to the growth of obligate halophiles and hyperthermophiles, respectively. Collectively, the detection of *Aminicenantes*-affiliated sequences across environmental extremes, coupled to their observed ubiquitous distribution on a global scale and the distinct patterns of community structure exhibited argues for a high level of intraphylum metabolic and adaptive diversity within the *Aminicenantes*. Therefore it is probable that *Aminicenantes* cells in nature exhibit multiple distinct metabolic capabilities, wide array of survival weapons, and various adaptive strategies. This, in turn, highlights the importance of obtaining multiple genomic assemblies that adequately represents the broad phylogenetic diversity of this phylum, as well as its wide environmental distribution to truly gauge the pangenomic diversity within the *Aminicenantes*. The recently acquired genomic information from single cell-based efforts from Sakinaw Lake represents admirable effort to investigate this understudied and yet-uncultured lineage. However, information from such assemblies should not be extrapolated to describe all members of the *Aminicenantes*. Indeed, the discovery of novel capabilities within well-establish lineages e.g. phototrophy amongst *Acidobacteria* [60], methane oxidation amongst the *Verrucomicrobia* [61], [62], anaerobic oxidation of ammonia amongst the *Planctomycetes* [63] highlights the importance of continued efforts to decipher and expand genomic diversity within various bacterial phyla.

## Supporting Information

File S1 **Contains the files: Figure S1** *Aminicenantes* relative abundance in different habitat types. **Figure S2** *Aminicenantes* relative abundance in response to various geochemical conditions. **Table S1** Summary of all high throughput-generated datasets analyzed in this study. **Table S2** List of all near full-length 16S rRNA sequences belonging to the *Aminicenantes* and their class/order level phylogenetic affiliations to *Aminicenantes*.
(DOC)
