# Supplementary material for: Global Patterns of Abundance, Diversity and Community Structure of the Aminicenantes (Candidate Phylum OP8)
Source: PLoS One. 2014 Mar 17;9(3):e92139. doi: 10.1371/journal.pone.0092139 (PMC3956909; doi:10.1371/journal.pone.0092139)
Supplement: File S1 — Contains the files: Figure S1 Aminicenantes relative abundance in different habitat types. Figure S2 Aminicenantes relative abundance in response to various geochemical conditions. Table S1 Summary of all high throughput-generated datasets analyzed in this study. Table S2 List of all near full-length 16S rRNA sequences belonging to the Aminicenantes and their class/order level phylogenetic affiliations to Aminicenantes. (DOC) [file pone.0092139.s001.doc]

**Supplementary document**

Global patterns of abundance, diversity and community structure of the *Aminicenantes* (Candidate phylum OP8)

**Authors:** Ibrahim F. Farag, James P. Davis, Noha H. Youssef, and Mostafa S. Elshahed

Figure S1 *Aminicenantes* relative abundance in different habitat types


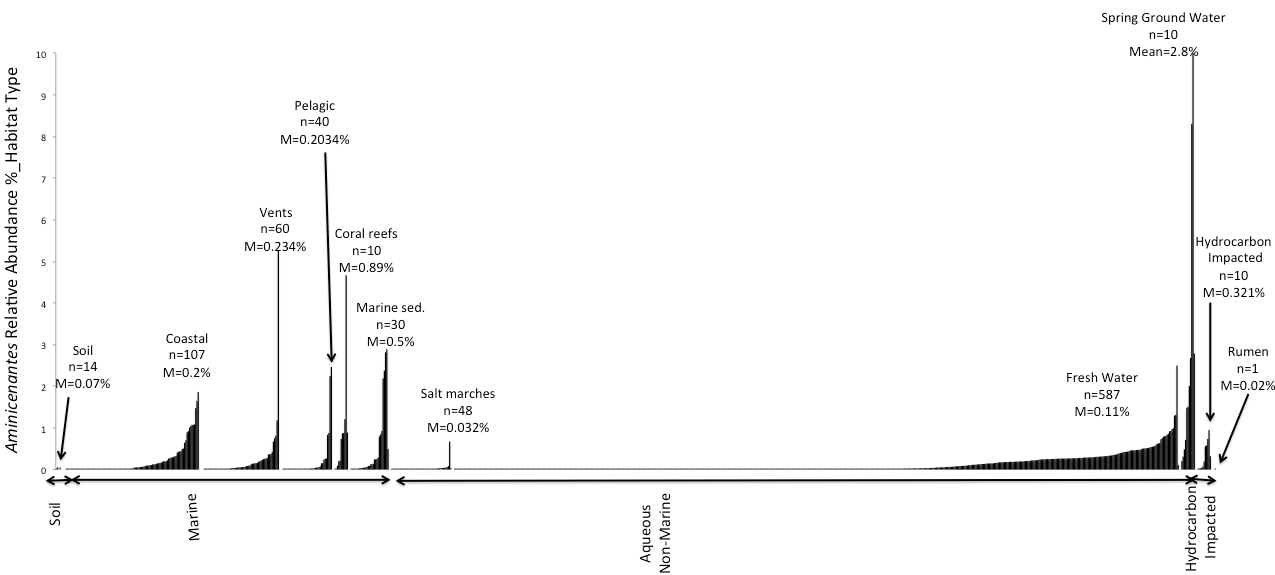


Figure S2 *Aminicenantes* relative abundance in response to various geochemical conditions

a) Temperature


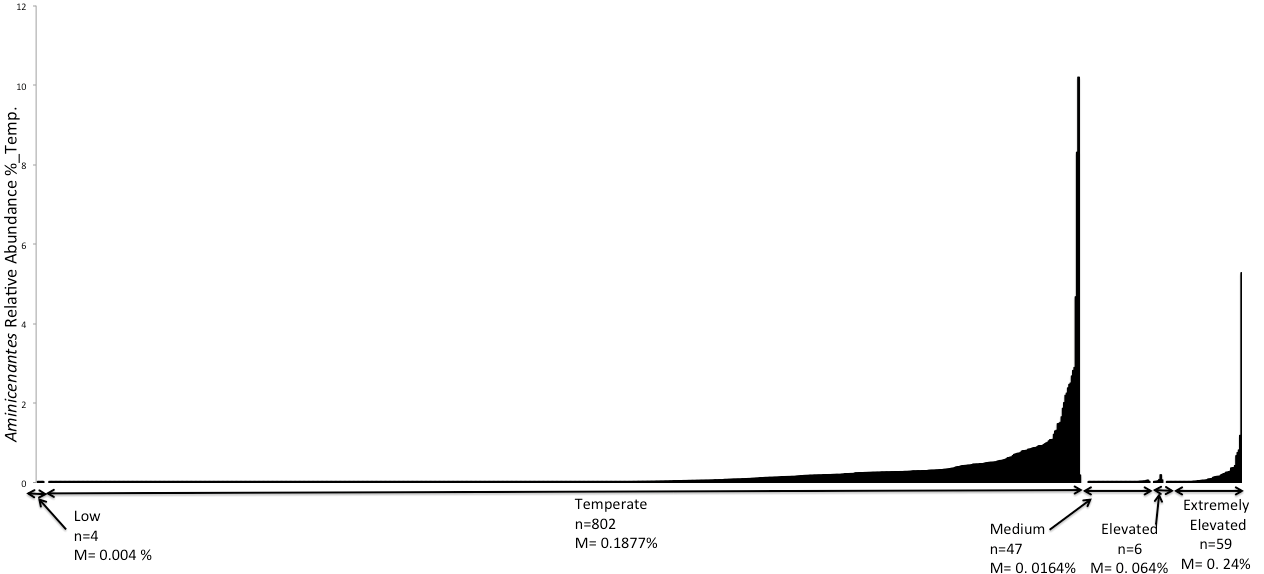


b) Oxygen tension


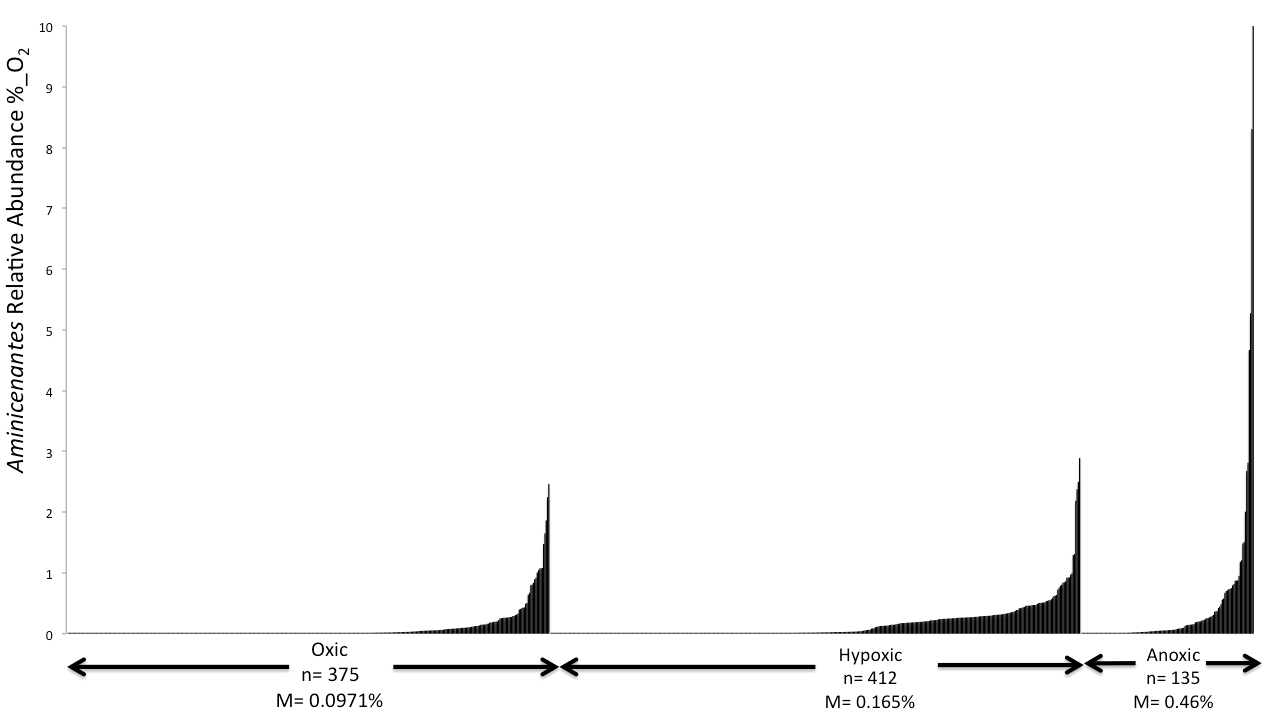


c) Salinity


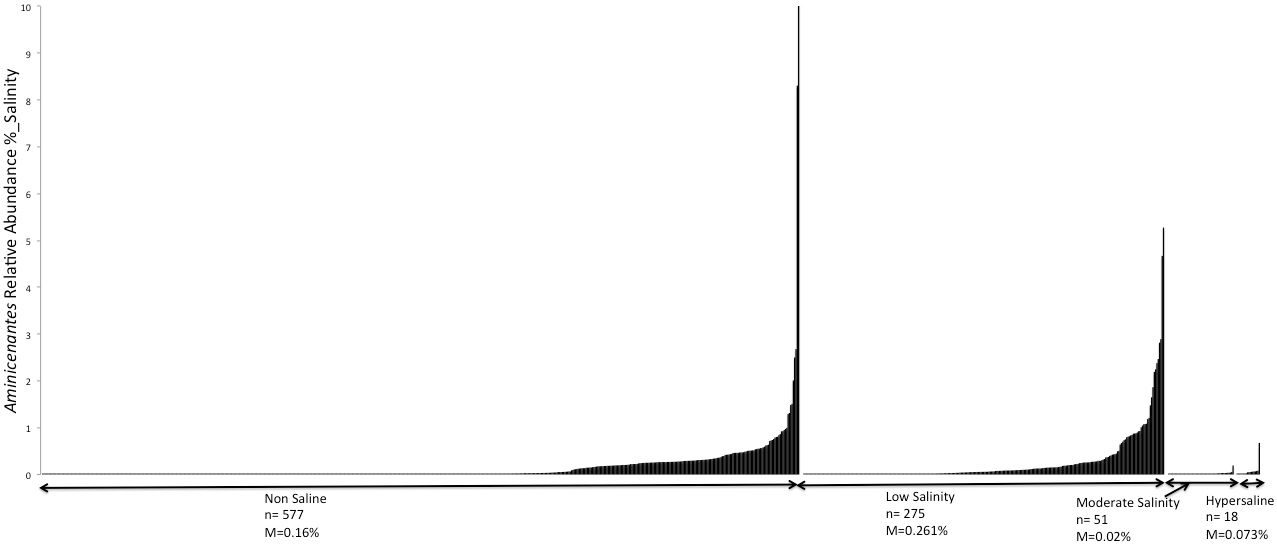


Table S1 Summary of all high throughput-generated datasets analyzed in this study.

|  | Project Name | Datasets ID | Number of datasets | Datasets with *Aminicenantes* | *Aminicenantes* Abundance % | Project Description | Temperature | Salinity | Oxygen tension | Ref. |
| --- | --- | --- | --- | --- | --- | --- | --- | --- | --- | --- |
| Marine |  |  |  |  |  |  |  |  |  |  |
| Coastal |  |  |  |  |  |  |  |  |  |  |
|  | ICoMM Cooperative Run | LCR_0001 | 4 | 4 | 0.016± 0.005 | Microbial diversity in coastal systems along a latitudinal gradient from South Atlantic to the Caribbean | Temperate | Low salinity (marine salinity) | Oxic |  |
|  |  | - |  |  |  |  |  |  |  |  |
|  |  | LCR_0006 |  |  |  |  |  |  |  |  |
|  | CaporasoIlluminaPNAS2011_3prime | 4476910.3 | 36 | 12 | 0.118±0.217 | Marine sediment | Temperate | Low salinity (marine salinity) | Anoxic |  |
|  |  | - |  |  |  |  |  |  |  |  |
|  |  | 4477224.3 |  |  |  |  |  |  |  |  |
|  | Three sediment comparison_Mangarove biome Mai Po Hong Kong | 4490067.3 | 14 | 13 | 0.0275±0.024 | Intertidal sediment | Temperate | High salinity | Anoxic |  |
|  |  | - |  |  |  |  |  |  |  |  |
|  |  | 4490054.3 |  |  |  |  |  |  |  |  |
|  | Mount Hope Bay Winter and Summer | KCK_MHB_Bv6 | 24 | 0 | 0 | Coastal | Temperate with thermal pollution from water cooled power plant | Low salinity (marine salinity) | Oxic |  |
|  | Hood Canal Washington | ICM_HCW_Bv6 | 16 | 0 | 0 | A long, narrow glacial fjord located 80 miles West of Seattle, WA, USA, has been impacted by human activities primarily through eutrophication, or high terrestrial input of nutrients leading to periodic and sustained low dissolved oxygen concentrations. | Temperate | Low salinity (marine salinity) | Hypoxic |  |
|  | SAND | HS_122 | 15 | 12 | 0.23±0.266 | Coastal sand | Temperate | Low salinity (marine salinity) | Oxic |  |
|  |  | - |  |  |  |  |  |  |  |  |
|  |  | HS_139 |  |  |  |  |  |  |  |  |
|  | Frisian Island Sylt | FIS_0001 | 16 | 15 | 0.128±0.15 | Coastal Habitat | Temperate | Low salinity (marine salinity) | Oxic |  |
|  |  | - |  |  |  |  |  |  |  |  |
|  |  | FIS_0016 |  |  |  |  |  |  |  |  |
|  | English water channel PML | 4451488 | 12 | 1 | 0.008647527 | Coastal Habitat | Temperate | Low salinity (marine salinity) | Oxic |  |
|  |  | - |  |  |  |  |  |  |  |  |
|  |  | 4451502 |  |  |  |  |  |  |  |  |
|  | Coastal New England | CNE_0001 | 2 | 2 | 0.005±0.001 | Coastal Habitat | Temperate | Low salinity (marine salinity) | Oxic |  |
|  |  | - |  |  |  |  |  |  |  |  |
|  |  | CNE_0004 |  |  |  |  |  |  |  |  |
|  | L4 Timeseries | 4468001- 4468310 | 310 | 18 | 0.0016±0.00094 | Coastal water (English Channel L4 marine observatory) | Temperate | Low salinity (marine salinity) | Oxic |  |
|  | Western English Channel | PML_0002 | 2 | 2 | 0.006±0.001 | Coastal Habitat | Temperate | Low salinity (marine salinity) | Oxic |  |
|  |  | - |  |  |  |  |  |  |  |  |
|  |  | PML_63 |  |  |  |  |  |  |  |  |
|  | Humboldt Marine Ecosystem | VAG_0001 | 16 | 16 | 0.15±0.1 | The West Coast of South America from Northern Peru to the southern tip of Chile | Temperate | Low salinity (marine salinity) | Oxic |  |
|  |  | - |  |  |  |  |  |  |  |  |
|  |  | VAG_0016 |  |  |  |  |  |  |  |  |
|  | OTUs Variation with Depth | HOT201_770 | 1 | 1 | 0.00262 | Subtidal sands of the German Wadden Sea | Temperate | Low salinity (marine salinity) | Oxic |  |
|  | Microbial diversity and function from Northeast Brazil mangrove sediments | SRR171663 | 5 | 1 | 0.00044 | Three distinct mangroves located on the coast of São Paulo State, Brazil | Temperate | Low salinity (marine salinity) | Anoxic |  |
|  |  | - |  |  |  |  |  |  |  |  |
|  |  | SRR038651 |  |  |  |  |  |  |  |  |
|  | Mangrove environments in Guanabara Bay (Rio de Janeiro, Brazil) | SRR066494 | 12 | 12 | 1.0148±0.477 | Mangrove Habitat | Temperate | Low salinity (marine salinity) | Anoxic |  |
|  |  | - |  |  |  |  |  |  |  |  |
|  |  | SRR066509 |  |  |  |  |  |  |  |  |
|  | Microbial diversity and function from Northeast Brazil mangrove sediments | SRR171663 | 5 | 1 | 0.00044 | Three distinct mangroves located on the coast of São Paulo State, Brazil | Temperate | Low salinity (marine salinity) | Anoxic |  |
|  |  | - |  |  |  |  |  |  |  |  |
|  |  | SRR038651 |  |  |  |  |  |  |  |  |
|  | Rocky intertidal of the northeast Pacific | 4468543.3 | 50 | 5 | 0.0019±0.0014 | The coast of Washington state in 2009 and 2010, including 2 locales: Tatoosh Island and Second Beach | Temperate | Low salinity (marine salinity) | Oxic |  |
|  |  | - |  |  |  |  |  |  |  |  |
|  |  | 4468591.3 |  |  |  |  |  |  |  |  |
|  | Ocean Acidification | 4468738- 4468810 | 72 | 0 | 0 | Coastal waters of a fjord close to Bergen, Norway | Temperate | Low salinity (marine salinity) | Oxic |  |
| Hydrothermal vent |  |  |  |  |  |  |  |  |  |  |
|  | Lau Hydrothermal vent | ALR_0001 | 10 | 2 | 0.0145±0.04 | Hydrothermal Vent Ecosystem | Extremely elevated | Low salinity (marine salinity) | Anoxic |  |
|  |  | - |  |  |  |  |  |  |  |  |
|  |  | ALR_0019 |  |  |  |  |  |  |  |  |
|  | Black Smokers | 4483600 | 64 | 32 | 0.21±0.25 | Vent fields surveyed include deposits from the Mid-Atlantic Ridge, Eastern Lau Spreading Center and Guayamas Basin | Extremely elevated | Low salinity (marine salinity) | Anoxic |  |
|  |  | - |  |  |  |  |  |  |  |  |
|  |  | 4483663 |  |  |  |  |  |  |  |  |
|  | Azorian Shallow Vents | ASV_0001 | 1 | 1 | 0.024 | Hydrothermal Vent Ecosystem | Extremely elevated | Low salinity (marine salinity) | Anoxic |  |
|  | Azores Water Project | AWP_0003 | 1 | 1 | 0.003 | Hydrothermal Vent Ecosystem | Extremely elevated | Low salinity (marine salinity) | Anoxic |  |
|  | Pilot Seamounts | FS312 | 2 | 2 | 0.0091±0.00157 | Hydrothermal Vent Ecosystem | Extremely elevated | Low salinity (marine salinity) | Anoxic |  |
|  |  | - |  |  |  |  |  |  |  |  |
|  |  | FS396 |  |  |  |  |  |  |  |  |
|  | Diffuse flow hydrothermal fluids from seamounts | FS389 | 12 | 12 | 0.0358±0.0671 | Hydrothermal Vent Ecosystem | Extremely elevated | Low salinity (marine salinity) | Anoxic |  |
|  |  | - |  |  |  |  |  |  |  |  |
|  |  | FS521 |  |  |  |  |  |  |  |  |
|  | Deep-sea hydrothermal vents | SRR091821 | 4 | 3 | 0.0035±0.005 | Hydrothermal Vent Ecosystem | Extremely elevated | Low salinity (marine salinity) | Anoxic |  |
|  |  | - |  |  |  |  |  |  |  |  |
|  |  | SRR192300 |  |  |  |  |  |  |  |  |
|  | Deep-sea vent sulfide deposits | SRR027255 | 2 | 2 | 0.034±0.025 | Hydrothermal Vent Ecosystem | Medium | Low salinity (marine salinity) | Oxic |  |
|  |  | - |  |  |  |  |  |  |  |  |
|  |  | SRR027258 |  |  |  |  |  |  |  |  |
|  | Lost City_Archaeal Microevolution ICM_LCY_Bv6 | ICM_LCY_Bv6 | 4 | 0 | 0 | High-temperature vent environments | Medium | Low salinity (marine salinity) | Anoxic |  |
|  | Ocean Flows Through Sulfide and Basalt | KCK_SBF_Bv6 | 1 | 0 | 0 | Hydrothermal vent | Extremely elevated | Low salinity (marine salinity) | Anoxic |  |
| Pelagic |  |  |  |  |  |  |  |  |  |  |
|  | New Zealand Sediment | NZS_0001 | 16 | 15 | 0.0304±0.056 | The first project is to map benthic habitats and marine biodiversity of the seabed down to water depths of 1200 m in two strongly contrasted areas - the Chatham Rise and Challenger Plateau | Temperate | Low salinity (marine salinity) | Hypoxic |  |
|  |  | - |  |  |  |  |  |  |  |  |
|  |  | NZS_0016 |  |  |  |  |  |  |  |  |
|  | North Atlantic Deep Water Flow-Public Data | KCK_NADP_Bv6 | 6 | 0 | 0 | North Atlantic Deep Water (subsurface water) | Temperate | Low salinity (marine salinity) | Hypoxic |  |
|  | North Atlantic Deep Water Flow | KCK_NADW_Bv6 | 42 | 0 | 0 | North Atlantic Deep Water | Temperate | Low salinity (marine salinity) | Hypoxic |  |
|  | Black Sea Redox | BSR_0003 | 1 | 1 | 0.0085 | Anoxic marine habitat | Temperate | Low salinity (marine salinity) | Anoxic |  |
|  | Cariaco Basin | CAR_0001 | 14 | 13 | 0.11±0.223 | Anoxic marine habitat | Temperate | Low salinity (marine salinity) | Anoxic |  |
|  |  | - |  |  |  |  |  |  |  |  |
|  |  | CAR_0015 |  |  |  |  |  |  |  |  |
|  | Equatorial and north Pacific | EQP_1_34M | 5 | 5 | 1.195±0.98 | Marine Habitat | Temperate | Low salinity (marine salinity) | Oxic |  |
|  |  | - |  |  |  |  |  |  |  |  |
|  |  | EQP_1_SEC6 |  |  |  |  |  |  |  |  |
|  | North Atlantic Ocean | SRR029089 | 1 | 1 | 0.0024 | From subsurface (100 m depth), meso- (200-1000 m depth) and bathy-pelagic water masses (below 1000 m depth) of the North Atlantic Ocean | Temperate | Low salinity (marine salinity) | Oxic/Anoxic |  |
|  | Vertical stratification of microbial communities in the Red Sea revealed by 16S rDNA pyrosequencing | SRR043583  -  SRR070071 | 2 | 0 | 0 | Marine Habitat | Temperate | Low salinity (marine salinity) | Oxic |  |
|  | Arctic Ocean | 4469114 | 129 | 3 | 0.00002± 0.0002 | Arctic Ocean Acidification | Low | Low salinity (marine salinity) | Oxic |  |
|  |  | - |  |  |  |  |  |  |  |  |
|  |  | 4469246 |  |  |  |  |  |  |  |  |
|  | Amundsen Sea, Antarctica | SRR027287  -  SRR027300 | 14 | 0 | 0 | Low Temperature Marine Habitat | Low | Low salinity (marine salinity) | Oxic |  |
|  | Arctic Ocean in Summer and Winter | SRR027227- SRR027242 | 16 | 0 | 0 | Low Temperature Marine Habitat | Low | Low salinity (marine salinity) | Oxic |  |
|  | Arctic Chuki Beaufort | ACB_001- ACB_0016 | 11 | 0 | 0 | Pelagic | Low | Low salinity (marine salinity) | Oxic |  |
|  | Atlantic Ocean Transect | AOT_0001-AOT_0016 | 16 | 0 | 0 | Pelagic (North Atlantic) | Temperate | Low salinity (marine salinity) | Oxic |  |
|  | Amundsen Sea Antractica | ASA_0001-ASA_0014 | 14 | 0 | 0 | Amundsen sea (Antarctic) | Low | Low salinity (marine salinity) | Oxic |  |
|  | Blanes Bay Microbial Observatory | BMO_0001- BMO_0016 | 16 | 0 | 0 | NW Mediterranean Sea (pelagic) | Temperate | Low salinity (marine salinity) | Oxic |  |
|  | Baltic sea Proper | BSP_0001- BSP_0010 | 8 | 0 | 0 | Covers the part of the Baltic Sea, from Åland Sea to the Danish sounds (pelagic) | Temperate | Low salinity (marine salinity) | Oxic |  |
|  | Census Antractic Marine | CAM_0001- CAM_0016 | 16 | 0 | 0 | Pelagic marine | Low | Low salinity (marine salinity) | Oxic |  |
|  | Coastal Microbial Mats | CMM_0001- CMM_0009 | 9 | 0 | 0 | Antarctic Peninsula/Kerguelen Islands/Weddell Sea/Ross Sea | Low | Low salinity (marine salinity) | Oxic |  |
|  | Deep Arctic Ocean | DAO_0001- DAO_0016 | 13 | 0 | 0 | Three major oceanic basins of the Arctic Ocean: the Canada Basin, the Eurasian Basin and the Baffin Bay Basin. Samples were collected to target three different deep arctic water masses: the halocline, the Atlantic layer and the Baffin Bay intermediate water | Low | Low salinity (marine salinity) | Oxic |  |
|  | Gulf of Aqaba | GOA_0001- GOA_0016 | 16 | 0 | 0 | (Marine) pelagic | Temperate | Low salinity (marine salinity) | Oxic |  |
|  | ICoMM Cooperative Run | ICR_0001- ICR_0012 | 12 | 0 | 0 | Indian ocean (Marine pelagic) | Temperate | Low salinity (marine salinity) | Oxic |  |
|  | Helgoland Roads | MPI_0001-MPI_0016 | 16 | 0 | 0 | Marine (pelagic) | Temperate | Low salinity (marine salinity) | Oxic |  |
|  | South Pacific Gyre | KNX_0001- KNX_0008 | 8 | 0 | 0 | Pelagic | Temperate | Low salinity (marine salinity) | Oxic |  |
| Coral associated habitat |  |  |  |  |  |  |  |  |  |  |
|  | Mcav decontam stressed | 4455158 | 2 | 0 | 0 | Sponge Microbiome | Temperate | Low salinity (marine salinity) | Oxic |  |
|  |  | - |  |  |  |  |  |  |  |  |
|  |  | 4455159 |  |  |  |  |  |  |  |  |
|  | Coral Reef Sediment | CRS_0001 | 12 | 7 | 1.23±1.447 | Sponge Microbiome | Temperate | Low salinity (marine salinity) | Anoxic |  |
|  |  | - |  |  |  |  |  |  |  |  |
|  |  | CRS_0012 |  |  |  |  |  |  |  |  |
|  | Carribean Coral Bacteria | CCB_0006 | 1 | 1 | 0.004409074 | Sponge Microbiome | Temperate | Low salinity (marine salinity) | Oxic |  |
|  | Great Barrier Reef Sponge Bacteria | SPO_0016 | 1 | 1 | 0.004 | Sponge Microbiome | Temperate | Low salinity (marine salinity) | Oxic |  |
|  | Community structures of sub-seafloor microbes in coldwater coral carbonates | DRR001556 | 2 | 1 | 0.0996±0.0996 | Coral carbonates (Madrepora oculata and Lophelia pertusa) and the clay matrix microbiome | Temperate | Low salinity (marine salinity) | Anoxic |  |
|  |  | - |  |  |  |  |  |  |  |  |
|  |  | DRR001557 |  |  |  |  |  |  |  |  |
|  | Water of Palmyra | SRR001048 | 1 | 0 | 0 | Two atolls, Kingman and Palmyra, are part of the US national refuge system and have little or no local anthropogenic impacts | Temperate | Low salinity (marine salinity) | Oxic |  |
| Deep underground marine subsurface sediment |  |  |  |  |  |  |  |  |  |  |
|  | Station M Sediment | SMS_0008 | 5 | 5 | 0.0163±0.014 | Deep-sea time-series study in the abyssal ocean | Temperate | Low salinity (marine salinity) | Anoxic |  |
|  |  | - |  |  |  |  |  |  |  |  |
|  |  | SMS_0014 |  |  |  |  |  |  |  |  |
|  | Sediment from Peru Shelf and Basin | ODP_0002 | 7 | 6 | 0.62±0.931 | Marine Sediment | Temperate | Low salinity (marine salinity) | Anoxic |  |
|  |  | - |  |  |  |  |  |  |  |  |
|  |  | ODP_0016 |  |  |  |  |  |  |  |  |
|  | Sub-seafloor biosphere | SRR027356 | 5 | 5 | 0.693±0.8 | Sediment samples were collected between the 3rd and 10th March 2002 (Shipboard Scientific Party, 2003), mainly as advance piston cores using seawater as drilling fluid (Sites 1228 and 1229 on the Peru Margin) | Temperate | Low salinity (marine salinity) | Hypoxic |  |
|  |  | - |  |  |  |  |  |  |  |  |
|  |  | SRR027362 |  |  |  |  |  |  |  |  |
|  | Deep Subseafloor Sediment | CFU_0001 | 7 | 6 | 0.93±1.131 | Marine Sediment | Temperate | Low salinity (marine salinity) | Hypoxic |  |
|  |  | - |  |  |  |  |  |  |  |  |
|  |  | CFU_0011 |  |  |  |  |  |  |  |  |
|  | Three sediment | 4490068.3 | 8 | 8 | 0.0655±0.0326 | Marginal sea biome | Temperate | Low salinity (marine salinity) | Hypoxic |  |
|  |  | - |  |  |  |  |  |  |  |  |
|  |  | 4490068.3 |  |  |  |  |  |  |  |  |
| Aqueous Non-marine |  |  |  |  |  |  |  |  |  |  |
| Salt marshes |  |  |  |  |  |  |  |  |  |  |
|  | Spatial Scaling Diversity | SSD_0004 | 3 | 3 | 0.0023±0.00085 | Salt marsh sediment | Medium | Moderate salinity (5-15%) | Oxic |  |
|  |  | - |  |  |  |  |  |  |  |  |
|  |  | SSD_0008 |  |  |  |  |  |  |  |  |
|  | Salt Marsh Sediments (JLB_TIDE) | CH_Aug | 9 | 9 | 0.013±0.01 | Salt marsh sediment | Medium | Moderate salinity (5-15%) | Oxic |  |
|  |  | - |  |  |  |  |  |  |  |  |
|  |  | SW_Sept |  |  |  |  |  |  |  |  |
|  | Salt Marsh Sediments | TIDE_0002_CF2 | 10 | 10 | 0.014±0.009 | Salt marsh sediment | Medium | Moderate salinity (5-15%) | Oxic |  |
|  |  | - |  |  |  |  |  |  |  |  |
|  |  | West_Sept |  |  |  |  |  |  |  |  |
|  | Coastal wetlands in Yellow River Estuary | SRR205889 | 1 | 1 | 0.0113 | Estuary habitat | Medium | Moderate salinity (5-Estu15%) | Oxic |  |
|  | Saline and hypersaline sediment | SRR364695 | 4 | 4 | 0.22±0.27 | La Sal del Rey is a shallow (B1m depth), 215- hectare hypersaline lake located within the Lower Rio Grande Valley National Wildlife Refuge, Hidalgo County, TX | Temperate | Hypersaline | Oxic |  |
|  |  | - |  |  |  |  |  |  |  |  |
|  |  | SRR364704 |  |  |  |  |  |  |  |  |
|  | Salt marshes perturbed by nutrients | SRR070884 | 22 | 22 | 0.0187±0.012 | 2008 from La Sal del Rey’s shoreline and lakebed. | Medium | Moderate salinity (5-15%) | Oxic |  |
|  |  | - |  |  |  |  |  |  |  |  |
|  |  | SRR071118 |  |  |  |  |  |  |  |  |
|  | Eel Pond- Woods Hole MA | KCK_EEL_Bv6 | 1 | 0 | 0 | Salt Pond | Medium | Moderate salinity (5-15%) | Oxic |  |
|  | Little Sippewissett Marsh | KCK_LSM_Bv6 | 21 | 0 | 0 | Salt marsh |  | Moderate salinity (5-15%) | Oxic |  |
| Temperate freshwater |  |  |  |  |  |  |  | Moderate salinity (5-15%) |  |  |
|  | Peccia_Lab_MFC_Study | 4465374 | 1 | 1 | 0.00145 | Waste water | Temperate | Moderate salinity (5-15%) | Oxic |  |
|  | Sinkhole | SRR013408 | 1 | 1 | 0.147496968 | Water-filled limestone sinkholes in northeastern Mexico. | Temperate | (Limestone salinity) | Anoxic |  |
|  | Bacterial pathogen diversity in sewage | SRR026596 | 2 | 2 | 0.15±0.06 | Sewage | Temperate | Not saline | Oxic |  |
|  |  | - |  |  |  |  |  |  |  |  |
|  |  | SRR026597 |  |  |  |  |  |  |  |  |
|  | Amazon Guianas water | SRR027243 | 4 | 4 | 0.2±0.136 | Temperate fresh water river | Temperate | Low salinity (marine salinity) | Oxic |  |
|  |  | - |  |  |  |  |  |  |  |  |
|  |  | SRR027246 |  |  |  |  |  |  |  |  |
|  | Amazon-Guianas water-sediment | AGW_0001 | 4 | 4 | 0.213±0.144 | Coastal | Temperate | Not saline | Oxic |  |
|  |  | - |  |  |  |  |  |  |  |  |
|  |  | AGW_0004 |  |  |  |  |  |  |  |  |
|  | CaporasoIlluminaPNAS2011_3 prime | 4476753.3 | 20 | 20 | 0.1008±0.178 | Creek | Temperate | Low salinity (marine salinity) | Oxic |  |
|  |  | - |  |  |  |  |  |  |  |  |
|  |  | 4477215.3 |  |  |  |  |  |  |  |  |
|  | Three sediment comparison | 4490053.3 | 13 | 13 | 0.094±0.0303 | Freshwater habitat (Liuxi river reservoir) | Temperate | Low salinity (marine salinity) | Oxic |  |
|  |  | - |  |  |  |  |  |  |  |  |
|  |  | 4490041.3 |  |  |  |  |  |  |  |  |
|  | Columbia River coastal margin | SRR187794 | 1 | 1 | 0.0034 | Estuarine and shallow-water environments | Temperate | Low salinity (marine salinity) | Hypoxic |  |
|  | McMahon | 4462973.3 | 1511 | 537 | 0.1097±0.217 | Temperate Freshwater | Temperate | Non saline | Oxic/Anoxic |  |
|  |  | - |  |  |  |  |  |  |  |  |
|  |  | 4465210.3 |  |  |  |  |  |  |  |  |
|  | CaporasoIlluminaPNAS2011_3prime | 4476915.3 | 12 | 3 | 0.0004±0.001 | Fresh water lake | Temperate | Non saline | Oxic |  |
|  |  | - |  |  |  |  |  |  |  |  |
|  |  | 4477208.3 |  |  |  |  |  |  |  |  |
| Spring and ground water |  |  |  |  |  |  |  |  |  |  |
|  | GBS and SSW, in the US Great Basin | SRR074959 | 4 | 0 | 0 | Hot spring | Extremely elevated | Non saline | Oxic |  |
|  |  | - |  |  |  |  |  |  |  |  |
|  |  | SRR076609 |  |  |  |  |  |  |  |  |
|  | mladenov_bangladesh_arsenic | 4455878 | 20 | 10 | 1.4±2.75 | Ground water habitat heavily contaminated by arsenic | Temperate | Non saline | Anoxic |  |
|  |  | - |  |  |  |  |  |  |  |  |
|  |  | 4455898 |  |  |  |  |  |  |  |  |
|  | Hot Spring of colombian andes | SRR094470 | 1 | 0 | 0 | In-depth characterization via complementing culture-independent approaches of the microbial community in an acidic hot spring of the Colombian Andes. | Elevated | Non saline | Oxic |  |
| Soil |  |  |  |  |  |  |  |  |  |  |
| Frozen lands (permafrost) |  |  |  |  |  |  |  |  |  |  |
|  | Arctic LTER | Arctic LTER | 1 | 1 | 0.010406068 | Arctic LTER studies the ecology of the surrounding tundra, streams, and lakes | Low | Non saline | Oxic |  |
|  | Chu_Arctic | 4455826 | 49 | 0 | 0 | 29 heath tundra sites close to the top of exposed ridges in the Canadian, Alaskan and European Arctic in the summers of 2007 and 2008 | Low | Non saline | Oxic |  |
|  |  | - |  |  |  |  |  |  |  |  |
|  |  | 4455877 |  |  |  |  |  |  |  |  |
|  | Antarctic Dry Valleys | ERR055326 | 3 | 0 | 0 | McMurdo Dry Valleys compose the largest inland ice-free area of the Antarctic continent | Low | High salinity | Oxic |  |
|  |  | - |  |  |  |  |  |  |  |  |
|  |  | ERR055328 |  |  |  |  |  |  |  |  |
|  | Arctic soil | SRR036794 | 1 | 0 | 0 | Inland ice-free area of the Antarctic continent | Low | Non saline | Oxic |  |
| Arid and Semi-arid soils |  |  |  |  |  |  |  |  |  |  |
|  | Biogeography of biocrust soil bacteria | 4501995 | 41 | 0 | 0 | Cold-winter (continental) deserts and semideserts-desert/Colorado | Low | Non saline | Oxic |  |
|  |  | - |  |  |  |  |  |  |  |  |
|  |  | 4502036 |  |  |  |  |  |  |  |  |
|  | Semiarid soil above Kartchner Caverns | SRR090399-SRR139151 | 5 | 0 | 0 | Semiarid soils | Temperate | Non saline | Oxic |  |
| Grassland |  |  |  |  |  |  |  |  |  |  |
|  | Eilers_soils | 4455899- | 48 | 0 | 0 | Grassland, hardwood forest, and coniferous forest incubations with glucose, glycine, and citric acid | Temperate | Non saline | Oxic | [(Kathryn G. Eilers 2012)](#RANGE!_ENREF_30) |
|  |  | 4455948 |  |  |  |  |  |  |  |  |
|  | Icelandic critical zone | 4501863 | 9 | 0 | 0 | Temperate grasslands-subterrestrial habitat-volcanic soil-Skorradalur-Iceland | Temperate | Non saline | Oxic |  |
|  |  | - |  |  |  |  |  |  |  |  |
|  |  | 4505429 |  |  |  |  |  |  |  |  |
|  | CaporasoIlluminaPNAS2011_3 prime | 4476929.3 | 21 | 5 | 0.041±0.17 | Soil | Temperate | Non saline | Oxic |  |
|  |  | - |  |  |  |  |  |  |  |  |
|  |  | 4477220.3 |  |  |  |  |  |  |  |  |
|  | Grassland sites located in the German Biodiversity Exploratory Schwäbische Alb | SRR064380 | 2 | 2 | 0.0073±0.0007 | Grassland and forest | Temperate | Non saline | Oxic |  |
|  |  | - |  |  |  |  |  |  |  |  |
|  |  | SRR064371 |  |  |  |  |  |  |  |  |
|  | Hofmockel-Pool-6.KH20 | 4468477 | 60 | 3 | 0.000025 | Bacterial biodiversity between native and novel exotic-dominated communities exposed to irrigation treatment | Temperate | Non saline | Oxic |  |
|  |  | - |  |  |  |  |  |  |  |  |
|  |  | 4468537 |  |  |  |  |  |  |  |  |
| Agriculture |  |  |  |  |  |  |  |  |  |  |
|  | Ferromanganese paddy soil | 4502145 | 1 | 1 | 0.054867992 | Paddy field | Temperate | Non saline | Anoxic |  |
|  | Argentinean soils microbial diversity as revealed by 16s rRNA pyrosequencing | SRR090399 | 2 | 0 | 0 | Agricultural sites of La Pampa Ondulada region in Buenos Aires | Temperate | Non saline | Oxic |  |
|  |  | - |  |  |  |  |  |  |  |  |
|  |  | SRR095659 |  |  |  |  |  |  |  |  |
|  | Soil microbial diversity composition and structure under elevated Carbon dioxide | ERR024294-ERR024299 | 6 | 0 | 0 | Cedar Creek Ecosystem Science Reserve in Minnesota, USA (soil samples from 24 plots) (Artificial agricultural sites) | Temperate | Non saline | Oxic / with elevated CO2 levels |  |
|  | Rousk pH | 4455655.3 | 24 | 1 | 0-0.00024 | Soils collected across a long-term liming experiment (pH 4.0–8.3) | Temperate | Non saline | Oxic |  |
|  |  | - |  |  |  |  |  |  |  |  |
|  |  | 4455679.3 |  |  |  |  |  |  |  |  |
| Heavy metals and hydrocarbon contaminated soil |  |  |  |  |  |  |  |  |  |  |
|  | Bacterial community in two European soil responded to phenanthren spiking | 4491955 | 1 | 0 | 0 | To unravel the responses of microbial communities in soils and sediments to PAH pollution | Low | Non saline | Oxic |  |
|  | Utilizing Biphenyl, Benzoate, and Naphthalene in Long-Term Contaminated soil | ERR054049 | 7 | 1 | 0.00659261 | Soil contaminated with hydrocarbons | Temperate | Non saline | Oxic |  |
| Hydrocarbon Impacted environments |  |  |  |  |  |  |  |  |  |  |
|  | Zodletone | S1-S4 | 4 | 4 | 0.7078±0.157 | Petroleum | Temperate | Non saline | Anoxic |  |
|  | Alberta oil sands tailings pond | SRR090660 | 8 | 4 | 0.035±0.06 | Petroleum | Elevated | Moderate salinity (5-15%) | Anoxic/but have an Oxic surface layer |  |
|  |  | - |  |  |  |  |  |  |  |  |
|  |  | SRR090667 |  |  |  |  |  |  |  |  |
|  | Bulk Fluids and Biofilms of a North Slope Oil Facility | SRR065815 | 1 | 1 | 0.031126233 | Petroleum | Elevated | Moderate salinity (5-15%) | Anoxic |  |
|  | High-Temperature Continental Petroleum Reservoir/ Huabei Oilfield in China | SRR203252 | 1 | 1 | 0.073403474 | Petroleum | Elevated | Hypersaline | Anoxic |  |
| Herbivorous Gut |  |  |  |  |  |  |  |  |  |  |
|  | Nitrogen metabolism and rumen microbial enumeration | 4491447 | 3 | 0 | 0 | Rumen Ecosystem | Medium | Non saline | Anoxic |  |
|  |  | - |  |  |  |  |  |  |  |  |
|  |  | 4491449 |  |  |  |  |  |  |  |  |
|  | Rumen | 4483775 | 1 | 0 | 0 | Rumen Ecosystem | Medium | Non saline | Anoxic |  |
|  | CattleRumen | SRR019242 | 1 | 1 | 0.018339468 | Rumen Ecosystem | Medium | Non saline | Anoxic |  |
| OTHERS |  |  |  |  |  |  |  |  |  |  |
|  | Bacterial Ecology | 4479790 | 3 | 0 | 0 | Dust | Temperate | Non saline | Oxic |  |
|  |  | - |  |  |  |  |  |  |  |  |
|  |  | 4479792 |  |  |  |  |  |  |  |  |
|  | NEPRC African green | 4492964 | 15 | 0 | 0 | Mammalia associated habitat | Temperate | Non saline | Oxic |  |
|  |  | - |  |  |  |  |  |  |  |  |
|  |  | 4492979 |  |  |  |  |  |  |  |  |
|  | Bowers_storm peak_air | 4456075 | 9 | 0 | 0 | Atmospheric microbial abundance, community composition, and ice nucleation at a high-elevation site in northwestern Colorado | Low | Non saline | Oxic |  |
|  |  | - |  |  |  |  |  |  |  |  |
|  |  | 4456085 |  |  |  |  |  |  |  |  |

Table S2 List of all near full-length 16S rRNA sequences belonging to the *Aminicenantes* and their class/order level phylogenetic affiliations to *Aminicenantes*. Sequences identified in this study (as described in the materials and methods) are in bold.

| Classification Rank^1^ | Clone Name | GenBank Accession Number | Source | Reference |
| --- | --- | --- | --- | --- |
| P_-*Aminicenantes* |  |  |  |  |
| C-OP8_1 |  |  |  |  |
| O-Unclassified |  |  |  |  |
|  | Z17M64B | FJ484465.1 | Microbial autonomous rover world's deepest phreatic sinkhole wall biomat El Zacaton 17m depth |  |
|  | NA | AJ249097.1 | DCP-dechlorinating consortium |  |
|  | LP30MUD9 | FJ901651.1 | Phreatic limestone sinkholes northeastern Mexico biomat 30m deep cenote La Palita |  |
|  | GuBH2-AD-75 | AJ519671.1 | Uranium mill tailings soil sample |  |
|  | HS9-75 | AY221615.1 | Metal and hydrocarbon contaminated soil |  |
|  | **WCHA1-39** | **AF050552.1** | **Hydrocarbon- and chlorinated-solvent-contaminated aquifer** |  |
|  | **HS9-30** | **AY221610.1** | **Metal and hydrocarbon contaminated soil** |  |
| O-HMMV |  |  |  |  |
|  | Napoli-3B-31; BC07-3B-31 | AY592704.1 | Deep-sea mud volcano Napoli |  |
|  | HMMVPog-54 | AJ704718.1 | Marine sediment |  |
|  | FS142-68B-02 | AY704402.1 | Oceanic crust |  |
|  | ODP1230B20.28 | AB177172.1 | Seafloor methane hydrate |  |
|  | ODP1230B18.23 | AB177161.1 | Seafloor methane hydrate |  |
|  | GN01-8.012 | DQ154847.1 | Hypersaline Microbial Mat |  |
|  | VHS-B3-20 | DQ394934.1 | Harbor sediment |  |
|  | VHS-B3-2 | DQ394925.1 | [Harbor sediment clone VHS-B3-2](#RANGE!_ENREF_42) |  |
|  | SURF-GC205-Bac9 | DQ521812.1 | [Anaerobic Methane Oxidizing ANME-1b Archaea Hypersaline Sediments Gulf Mexico sediments clone SURF-GC205-Bac9](#RANGE!_ENREF_24) |  |
|  | SMI1-GC205-Bac3p | DQ521791.1 | [Anaerobic Methane Oxidizing ANME-1b Archaea Hypersaline Sediments Gulf Mexico sediments clone SMI1-GC205-Bac3p](#RANGE!_ENREF_24) |  |
|  | MSB-3A8 | DQ811949.1 | Mangrove soil |  |
|  | MSB-4A8 | DQ811948.1 | Mangrove soil |  |
|  | NKB18 | AB013270.1 | Deep sea sediment |  |
|  | MSB-5B7 | DQ811947.1 | Mangrove soil |  |
|  | KM22B-48 | AB300119.1 | Holocene mud sediment |  |
|  | 10bav_F8red | EU181473.1 | Continental margin marine sediments |  |
|  | MAT-CR-M3-E05 | EU245597.1 | Hypersaline microbial mat |  |
|  | MAT-CR-M1-C02 | EU245402.1 | Hypersaline microbial mat |  |
|  | 68_st3_10-12cm | EU290736.1 | Namibian upwelling system sediment 10-12cm depth |  |
|  | MAT-CR-H5-C05 | EU245280.1 | Hypersaline microbial mat |  |
|  | MAT-CR-M4-F08 | EU245679.1 | Hypersaline microbial mat |  |
|  | MAT-CR-P5-G05 | EU246246.1 | Hypersaline microbial mat |  |
|  | 8bav_B6_arb | EU181476.1 | Continental margin marine sediments |  |
|  | 10bav_H12red | EU181480.1 | Continental margin marine sediments |  |
|  | MAT-CR-H1-B08 | EU245054.1 | Hypersaline microbial mat |  |
|  | 8bav_A12_arb | EU181474.1 | Continental margin marine sediments |  |
|  | MAT-CR-H5-B04 | EU245272.1 | Hypersaline microbial mat |  |
|  | MD2896-B22 | EU048680.1 | Surface marine sediment Slope South China Sea |  |
|  | MAT-CR-M2-A02 | EU245470.1 | Hypersaline microbial mat |  |
|  | 5bav_C7arb | EU181475.1 | Continental margin marine sediments |  |
|  | MAT-CR-P3-E01 | EU246105.1 | Hypersaline microbial mat |  |
|  | LV-Bac24 | AM943576.1 | Lagoa Vermelha Brazil carbonaceous sediments hypersaline lagoon |  |
|  | MD2896-B54 | EU385676.1 | Stratified sediments Core MD05-2896 (subseafloor sediment South China Sea clone) |  |
|  | MD2898-B18 | EU386060.1 | Subseafloor sediment South China Sea |  |
|  | MD2902-B52 | EU385872.1 | Stratified sediments Core MD05-2896 (subseafloor sediment South China Sea clone) |  |
|  | MD2896-B195 | EU385778.1 | Stratified sediments Core MD05-2896 (subseafloor sediment South China Sea clone) |  |
|  | MD2896-B142 | EU385742.1 | Stratified sediments Core MD05-2896 (subseafloor sediment South China Sea clone) |  |
|  | MD2900-B2 | EU386077.1 | Subseafloor sediment South China Sea |  |
|  | MD2896-B58 | EU385679.1 | Stratified sediments Core MD05-2896 (subseafloor sediment South China Sea clone) |  |
|  | MD2896-B217 | EU385795.1 | Stratified sediments Core MD05-2896 (subseafloor sediment South China Sea clone) |  |
|  | MD2896-B153 | EU385753.1 | Stratified sediments Core MD05-2896 (subseafloor sediment South China Sea clone) |  |
|  | MD2902-B60 | EU385879.1 | Stratified sediments Core MD05-2896 (subseafloor sediment South China Sea clone) |  |
|  | MD2900-B20 | EU386094.1 | Subseafloor sediment South China Sea |  |
|  | MD2896-B261 | EU385820.1 | Stratified sediments Core MD05-2896 (subseafloor sediment South China Sea clone) |  |
|  | MD2898-B5 | EU386049.1 | Subseafloor sediment South China Sea |  |
|  | MD2902-B143 | EU385934.1 | Stratified sediments Core MD05-2902 (subseafloor sediment South China Sea clone) |  |
|  | 107A46 | EU735011.1 | Assemblages sediment station DBSE Northern Bering Sea |  |
|  | 107G57 | FJ416074.1 | Sediment station DBS1 Northern Bering Sea |  |
|  | JJB318 | GQ143790.1 | Yel Sea continental shelf sediment |  |
|  | JJB109 | GQ143758.1 | Yel Sea continental shelf sediment |  |
|  | JJB238 | GQ143784.1 | Yel Sea continental shelf sediment |  |
|  | JJB113 | GQ143760.1 | Yel Sea continental shelf sediment |  |
|  | JJB204 | GQ143767.1 | Yel Sea continental shelf sediment |  |
|  | SAW1_B101 | FJ716354.1 | Shal chemocline Bahamas Sawmill Sink column 10.3 m water depth |  |
|  | KZNMV-10-B13 | FJ712505.1 | (Kazan MV Sea) Kazan Mud Volcano Anaximander Mountains East Mediterranean Sea |  |
|  | CK_1C3_29 | EU488007.1 | Lucinid bivalve- symbiotic system: siliciclastic sedment Thalassia sea grass bed clone |  |
|  | bOHTK-35 | FJ873297.1 | Methane-rich cold seep located base sediments Okhotsk Sea |  |
|  | SSS65N | EU592441.1 | Salton Sea hypersaline sediment |  |
|  | CK_1C5_4 | EU488107.1 | lucinid bivalve- symbiotic system: siliciclastic sedment Thalassia sea grass bed clone |  |
|  | SSS34N | EU592427.1 | Salton Sea hypersaline sediment |  |
|  | CK_1C2_31 | EU487932.1 | lucinid bivalve- symbiotic system: siliciclastic sedment Thalassia sea grass bed clone |  |
|  | SPG12_343_353_B78 | FJ746154.1 | Oligotrophic marine sediments: South Pacific Gyre ocean sediment 5306 m water depth during Cruise Knox02rr |  |
|  | SPG12_461_471_B25 | FJ746322.1 | Oligotrophic marine sediments: South Pacific Gyre ocean sediment 5306 m water depth during Cruise Knox02rr |  |
|  | SPG12_461_471_B54 | FJ746185.1 | Oligotrophic marine sediments: South Pacific Gyre ocean sediment 5306 m water depth during Cruise Knox02rr | [(Durbin & Teske, 2011)](#RANGE!_ENREF_11) |
|  | BD72BR149 | GU363051.1 | Qiongdongnan basin Sea: marine sediment South China Sea |  |
|  | CK_1C4_64 | EU488093.1 | lucinid bivalve- symbiotic system: siliciclastic sedment Thalassia sea grass bed clone |  |
|  | B103B09 | FJ455883.1 | Sulfate-methane transition zone continental margin sediments (Santa Barbara Basin California) marine sediment |  |
|  | SPG12_213_223_B79 | FJ746232.1 | Oligotrophic marine sediments: South Pacific Gyre ocean sediment 5306 m water depth during Cruise Knox02rr | [(Durbin & Teske, 2011)](#RANGE!_ENREF_11) |
|  | SSS83N | EU592453.1 | Salton Sea hypersaline sediment |  |
|  | Ld1-5 | GQ246400.1 | China North Yel Sea sediments |  |
|  | Napoli-3B-43; BC07-3B-43 | AY592715.1 | Deep-sea mud volcano clone Napoli |  |
|  | **MERTZ_2CM_344** | **AF424327.1** | **Antarctic continental shelf sediment** |  |
|  | **ODP1230B20.07** | **AB177170.1** | **Subseafloor sediment at the Peru margin** |  |
|  | **ODP1230B11.12** | **AB177143.1** | **Subseafloor sediment at the Peru margin** |  |
|  | **KY1** | **AB116391.1** | **Marine sediment** |  |
|  | **K53** | **AB116395.1** | **Marine sediment** |  |
|  | **aquased50** | **DQ028266.1** | **Aquaculture pond sediment** |  |
|  | **Car60fc** | **AF224839.1** | **Anoxic zone of the Cariaco Basin** |  |
|  | **Y139** | **AB116432.1** | **Intensive shellfish aquaculture** |  |
|  | **MERTZ_21CM_147** | **AF424331.1** | **Antarctic continental shelf sediment** |  |
|  | **MERTZ_21CM_86** | **AF424330.1** | **Antarctic continental shelf sediment** |  |
|  | **MERTZ_2CM_346** | **AF424328.1** | **Antarctic continental shelf sediment** |  |
|  | **MERTZ_21CM_186** | **AF424326.1** | **Antarctic continental shelf sediment** |  |
|  | **Dover127** | **AY499864.1** | **Organically-enriched fish farm sediments** |  |
|  | **a2b031** | **AF419670.1** | **Hydrothermal sediments in the Guaymas Basin** |  |
| O-YNP |  |  |  |  |
|  | **OPB5** | **AF027067.1** | **Yellowstone hot spring** |  |
|  | **OPB23** | **AF027068.1** | **Yellowstone hot spring** |  |
|  | **OPS19A** | **AF027069.1** | **Yellowstone hot spring** |  |
|  | **OPS37** | **AF027070.1** | **Yellowstone hot spring** |  |
|  | **OBPB62** | **AY193076.1** | **Obsidian Pool** |  |
|  | **OBPB75** | **AY193081.1** | **Obsidian Pool** |  |
|  | **OBPB82** | **AY193084.1** | **Obsidian Pool** |  |
| O-OPB95 |  |  |  |  |
|  | OPB95 | AF027060.1 | Yellowstone hot spring |  |
|  | TP11 | EF198038.1 | Anaerobic thermophilic phenol-degrading enrichment |  |
|  | TP131 | EF205573.1 | Central Tibet geothermal spring mat |  |
|  | DTM39 | EF205501.1 | Central Tibet geothermal spring mat |  |
|  | 32b04 | EF515679.1 | Electricigen Enrichment MFC full-scale anaerobic bioreactor sludge treating brewery waste |  |
|  | 32b11 | EF515684.1 | Electricigen Enrichment MFC full-scale anaerobic bioreactor sludge treating brewery waste |  |
|  | 70b | FJ461971.1 | Industrial digestor mesophilic anaerobic reactor fed effluent chemical industry |  |
|  | 29b | FJ461970.1 | Industrial digestor mesophilic anaerobic reactor fed effluent chemical industry |  |
|  | 65a | FJ461968.1 | Industrial digestor mesophilic anaerobic reactor fed effluent chemical industry |  |
|  | 77a | FJ461969.1 | Industrial digestor mesophilic anaerobic reactor fed effluent chemical industry |  |
|  | TTA_B3 | AY297963.1 | Thermophilic anaerobic terephthalate-degrading sludge |  |
|  | **OBPB60** | **AY193120.1** | **Obsidian Pool** |  |
|  | **TUG23** | **AB011351.1** | **Thermophilic UASB granule (TUG)** |  |
|  | **TTA_H29** | **AY661412.1** | **Anaerobic hybrid reactor** |  |
|  | **OBPB32** | **AY193124.1** | **Obsidian Pool** |  |
|  | **OPS35B** | **AF027061.1** | **Obsidian Pool** |  |
|  | **UASB_TL56** | **AF254405.1** | **4-methylbenzoate-degrading methanogenic consortium** |  |
|  | **OPT3** | **AF027066.1** | **Yellowstone hot spring** |  |
|  | **OPS150** | **AF027064.1** | **Yellowstone hot spring** |  |
|  | **OPS88** | **AF027062.1** | **Yellowstone hot spring** |  |
|  | **OPS12** | **AF027065.1** | **Yellowstone hot spring** |  |
|  | **NA** | **AF402984.1** | **New Zealand: Rotorua, Kuirau Park** |  |
|  | **OBPB78** | **AY193121.1** | **Obsidian Pool** |  |
| O-SHA-124 |  |  |  |  |
|  | pLW-103 | DQ067009.1 | Lake Washington sediment |  |
|  | mbI-b45 | AB426227.1 | Anaerobic benzene degrading enrichment (lotus field soil) |  |
|  | mbI-b49 | AB426231.1 | Anaerobic benzene degrading enrichment (lotus field soil) |  |
|  | PS-Ba22 | EU399664.1 | Full-scale granular activated carbon anaerobic reactor phenol-degrading sludge |  |
|  | mbI-B25 | AB426209.1 | Anaerobic benzene degrading enrichment (lotus field soil) |  |
|  | mbI-B24 | AB426208.1 | Anaerobic benzene degrading enrichment (lotus field soil) |  |
|  | SWADLP3-22 | FJ535533.1 | Swine wastewater anaerobic UASB reactor |  |
|  | 6S1-13 | GU208248.1 | Dongping Lake sediment |  |
|  | A08-148-BAC | GQ340271.1 | Greece water column |  |
|  | SHA-124 | AJ306781.1 | DCP-dechlorinating consortium |  |
|  | S15B-MN72 | AJ583209.1 | Radioactive site ground water |  |
|  | **WCHA1-83** | **AF050553.1** | **Hydrocarbon- and chlorinated-solvent-contaminated aquifer** |  |
| C-OP8_2 |  |  |  |  |
|  | 7_st5_0-2cm | EU290738.1 | Namibian upwelling system sediment 0-2cm depth |  |
|  | 104B344 | EF687277.1 | (Nile Mediterranean) sediment underneath sulfide-oxidizing mat Chefren mud volcano Nile Deep Sea Fan Eastern Mediterranean |  |
|  | 4C1_cons | EF688191.1 | Anaerobic wastewater treatment system |  |
|  | LPBBBM52 | FJ902035.1 | Phreatic limestone sinkholes northeastern Mexico biomat sediment cenote La Palita |  |
|  | LT-SB-B88 | FJ755774.1 | Lake Taihu sediment |  |
|  | R76-B102 | AF449263.1 | Riftia pachyptila's tube |  |
|  | C1_B017 | AF419691.1 | Guaymas Basin hydrothermal sediment |  |
|  | **Dpcom247** | **AY453258** | **Bheri soil** |  |
|  | **WFeA1-35** | **AF050554.1** | **Contaminated aquifer** |  |
|  | **MERTZ_2CM_108** | **AF424325.1** | **Antarctic continental shelf sediment** |  |
|  | **MERTZ_21CM_72** | **AF424329.1** | **Antarctic continental shelf sediment** |  |
|  | **B02R006** | **AY197394.1** | **Guaymas Basin hydrothermal vent sediments** |  |
| C-OP8_3 |  |  |  |  |
|  | **a2b010** | **AF419671.1** | **Guaymas Basin** |  |
| C-Unclassified |  |  |  |  |
|  |  |  |  |  |
|  | 009E07_B_SD_P15 | CR933160.1 | Evry municipal wastewater treatment plant |  |
|  | 054H04_B_DI_P58 | CT574045.1 | Evry municipal wastewater treatment plant |  |
|  | 012H02_B_SD_P15 | CT573893.1 | Evry municipal wastewater treatment |  |
|  | NA | CU920186.1 | Mesophilic anaerobic digester which treats municipal wastewater sludge |  |

^1^ P= Phylum; C= Class; O= Order

**References**

1. Zinger L, Amaral-Zettler LA, Fuhrman JA, Horner-Devine MC, Huse SM, et al. (2011) Global patterns of bacterial beta-diversity in seafloor and seawater ecosystems. PLoS One 6: e24570.

2. Caporaso JG, Lauber CL, Walters WA, Berg-Lyons D, Lozupone CA, et al. (2011) Global patterns of 16S rRNA diversity at a depth of millions of sequences per sample. Proc Natl Acad Sci U S A 108 Suppl 1: 4516-4522.

3. Wang Y, Sheng HF, He Y, Wu JY, Jiang YX, et al. (2012) Comparison of the levels of bacterial diversity in freshwater, intertidal wetland, and marine sediments by using millions of illumina tags. Appl Environ Microbiol 78: 8264-8271.

4. Flores GE, Campbell JH, Kirshtein JD, Meneghin J, Podar M, et al. (2011) Microbial community structure of hydrothermal deposits from geochemically different vent fields along the Mid-Atlantic Ridge. Environ Microbiol 13: 2158-2171.

5. Dinsdale EA, Pantos O, Smriga S, Edwards RA, Angly F, et al. (2008) Microbial ecology of four coral atolls in the Northern Line Islands. PLoS One 3: e1584.

6. Webster G, Parkes RJ, Cragg BA, Newberry CJ, Weightman AJ, et al. (2006) Prokaryotic community composition and biogeochemical processes in deep subseafloor sediments from the Peru Margin. FEMS Microbiol Ecol 58: 65-85.

7. Hollister EB, Engledow AS, Hammett AJ, Provin TL, Wilkinson HH, et al. (2010) Shifts in microbial community structure along an ecological gradient of hypersaline soils and sediments. ISME J 4: 829-838.

8. Sahl JW, Gary MO, Harris JK, Spear JR (2011) A comparative molecular analysis of water-filled limestone sinkholes in north-eastern Mexico. Environ Microbiol 13: 226-240.

9. Roegner GC, Needoba JA, Baptista AM (2011) Coastal upwelling supplies oxygen-depleted water to the Columbia River estuary. PLoS One 6: e18672.

10. Murphy CN, Dodsworth JA, Babbitt AB, Hedlund BP (2013) Community microrespirometry and molecular analyses reveal a diverse energy economy in Great Boiling Spring and Sandy's Spring West in the U.S. Great Basin. Appl Environ Microbiol 79: 3306-3310.

11. Legg TM, Zheng Y, Simone B, Radloff KA, Mladenov N, et al. (2012) Carbon, metals, and grain size correlate with bacterial community structure in sediments of a high arsenic aquifer. Front Microbiol 3: 82.

12. Bohorquez LC, Delgado-Serrano L, Lopez G, Osorio-Forero C, Klepac-Ceraj V, et al. (2012) In-depth characterization via complementing culture-independent approaches of the microbial community in an acidic hot spring of the Colombian Andes. Microb Ecol 63: 103-115.

13. Chu H, Fierer N, Lauber CL, Caporaso JG, Knight R, et al. (2010) Soil bacterial diversity in the Arctic is not fundamentally different from that found in other biomes. Environ Microbiol 12: 2998-3006.

14. Lee CK, Barbier BA, Bottos EM, McDonald IR, Cary SC (2012) The Inter-Valley Soil Comparative Survey: the ecology of Dry Valley edaphic microbial communities. ISME J 6: 1046-1057.

15. Schutte UM, Abdo Z, Foster J, Ravel J, Bunge J, et al. (2010) Bacterial diversity in a glacier foreland of the high Arctic. Mol Ecol 19 Suppl 1: 54-66.

16. Kuske CR, Yeager CM, Johnson S, Ticknor LO, Belnap J (2012) Response and resilience of soil biocrust bacterial communities to chronic physical disturbance in arid shrublands. ISME J 6: 886-897.

17. Ortiz M, Neilson JW, Nelson WM, Legatzki A, Byrne A, et al. (2013) Profiling bacterial diversity and taxonomic composition on speleothem surfaces in Kartchner Caverns, AZ. Microb Ecol 65: 371-383.

18. Nacke H, Thurmer A, Wollherr A, Will C, Hodac L, et al. (2011) Pyrosequencing-based assessment of bacterial community structure along different management types in German forest and grassland soils. PLoS One 6: e17000.

19. O'Halloran LR, Borer ET, Seabloom EW, MacDougall AS, Cleland EE, et al. (2013) Regional contingencies in the relationship between aboveground biomass and litter in the world's grasslands. PLoS One 8: e54988.

20. He Z, Xu M, Deng Y, Kang S, Kellogg L, et al. (2010) Metagenomic analysis reveals a marked divergence in the structure of belowground microbial communities at elevated CO2. Ecol Lett 13: 564-575.

21. Rousk J, Baath E, Brookes PC, Lauber CL, Lozupone C, et al. (2010) Soil bacterial and fungal communities across a pH gradient in an arable soil. ISME J 4: 1340-1351.

22. Ding GC, Heuer H, Zuhlke S, Spiteller M, Pronk GJ, et al. (2010) Soil type-dependent responses to phenanthrene as revealed by determining the diversity and abundance of polycyclic aromatic hydrocarbon ring-hydroxylating dioxygenase genes by using a novel PCR detection system. Appl Environ Microbiol 76: 4765-4771.

23. Uhlik O, Wald J, Strejcek M, Musilova L, Ridl J, et al. (2012) Identification of bacteria utilizing biphenyl, benzoate, and naphthalene in long-term contaminated soil. PLoS One 7: e40653.

24. Youssef NH, Couger MB, Elshahed MS (2010) Fine-scale bacterial beta diversity within a complex ecosystem (Zodletone Spring, OK, USA): the role of the rare biosphere. PLoS One 5: e12414.

25. Saidi-Mehrabad A, He Z, Tamas I, Sharp CE, Brady AL, et al. (2013) Methanotrophic bacteria in oilsands tailings ponds of northern Alberta. ISME J 7: 908-921.

26. Stevenson BS, Drilling HS, Lawson PA, Duncan KE, Parisi VA, et al. (2011) Microbial communities in bulk fluids and biofilms of an oil facility have similar composition but different structure. Environ Microbiol 13: 1078-1090.

27. Li H, Yang SZ, Mu BZ, Rong ZF, Zhang J (2006) Molecular analysis of the bacterial community in a continental high-temperature and water-flooded petroleum reservoir. FEMS Microbiol Lett 257: 92-98.

28. Bowers RM, Lauber CL, Wiedinmyer C, Hamady M, Hallar AG, et al. (2009) Characterization of airborne microbial communities at a high-elevation site and their potential to act as atmospheric ice nuclei. Appl Environ Microbiol 75: 5121-5130.

29. Schlotelburg C, von Wintzingerode F, Hauck R, Hegemann W, Gobel UB (2000) Bacteria of an anaerobic 1,2-dichloropropane-dechlorinating mixed culture are phylogenetically related to those of other anaerobic dechlorinating consortia. Int J Syst Evol Microbiol 50 Pt 4: 1505-1511.

30. Joynt J, Bischoff M, Turco R, Konopka A, Nakatsu CH (2006) Microbial community analysis of soils contaminated with lead, chromium and petroleum hydrocarbons. Microb Ecol 51: 209-219.

31. Dojka MA, Hugenholtz P, Haack SK, Pace NR (1998) Microbial diversity in a hydrocarbon- and chlorinated-solvent-contaminated aquifer undergoing intrinsic bioremediation. Appl Environ Microbiol 64: 3869-3877.

32. Losekann T, Knittel K, Nadalig T, Fuchs B, Niemann H, et al. (2007) Diversity and abundance of aerobic and anaerobic methane oxidizers at the Haakon Mosby Mud Volcano, Barents Sea. Appl Environ Microbiol 73: 3348-3362.

33. Huber JA, Johnson HP, Butterfield DA, Baross JA (2006) Microbial life in ridge flank crustal fluids. Environ Microbiol 8: 88-99.

34. Inagaki F, Nunoura T, Nakagawa S, Teske A, Lever M, et al. (2006) Biogeographical distribution and diversity of microbes in methane hydrate-bearing deep marine sediments on the Pacific Ocean Margin. Proc Natl Acad Sci U S A 103: 2815-2820.

35. Dillon JG, Miller S, Bebout B, Hullar M, Pinel N, et al. (2009) Spatial and temporal variability in a stratified hypersaline microbial mat community. FEMS Microbiol Ecol 68: 46-58.

36. Zhang W, Ki JS, Qian PY (2008) Microbial diversity in polluted harbor sediments I: Bacterial community assessment based on four clone libraries of 16S rDNA. Estuarine Coastal and Shelf Science 76: 668-681.

37. Lloyd KG, Lapham L, Teske A (2006) An anaerobic methane-oxidizing community of ANME-1b archaea in hypersaline Gulf of Mexico sediments. Appl Environ Microbiol 72: 7218-7230.

38. Lina Li JG, Peter Nichols, Pierre Henry, Miki Yanagibayashi, Chiaki Kato (October 1999) Microbial Diversity in Nankai Trough Sediments at a Depth of 3,843 m. Journal of oceanography. Dordrecht: Kluwer Academic Publishers. pp. 635-642.

39. Takeuchi M, Komai T, Hanada S, Tamaki H, Tanabe S, et al. (2009) Bacterial and Archaeal 16S rRNA Genes in Late Pleistocene to Holocene Muddy Sediments from the Kanto Plain of Japan. Geomicrobiology Journal 26: 104-118.

40. Harrison BK, Zhang H, Berelson W, Orphan VJ (2009) Variations in archaeal and bacterial diversity associated with the sulfate-methane transition zone in continental margin sediments (Santa Barbara Basin, California). Appl Environ Microbiol 75: 1487-1499.

41. Isenbarger TA, Finney M, Rios-Velazquez C, Handelsman J, Ruvkun G (2008) Miniprimer PCR, a new lens for viewing the microbial world. Appl Environ Microbiol 74: 840-849.

42. Li T, Wang P (2013) Biogeographical distribution and diversity of bacterial communities in surface sediments of the South China Sea. J Microbiol Biotechnol 23: 602-613.

43. Yinxin Zeng YZ, Bo Chen, Jacqueline M. Grebmeier, Huirong Li, Yong Yu, Tianling Zheng (June 2011) Phylogenetic diversity of sediment bacteria in the northern Bering Sea. Heidelberg: Springer-Verlag. pp. 907-919.

44. Hong JK, Cho JC (2012) High level of bacterial diversity and novel taxa in continental shelf sediment. J Microbiol Biotechnol 22: 771-779.

45. Pachiadaki MG, Lykousis V, Stefanou EG, Kormas KA (2010) Prokaryotic community structure and diversity in the sediments of an active submarine mud volcano (Kazan mud volcano, East Mediterranean Sea). FEMS Microbiol Ecol 72: 429-444.

46. Jesse G. Dillon LMM, Amber L. Trout (October 2009) Seasonal changes in bacterial diversity in the Salton Sea. Hydrobiologia. Dordrecht: Kluwer Academic Publishers. pp. 49-64.

47. Durbin AM, Teske A (2011) Microbial diversity and stratification of South Pacific abyssal marine sediments. Environ Microbiol 13: 3219-3234.

48. Bowman JP, McCuaig RD (2003) Biodiversity, community structural shifts, and biogeography of prokaryotes within Antarctic continental shelf sediment. Appl Environ Microbiol 69: 2463-2483.

49. Asami H, Aida M, Watanabe K (2005) Accelerated sulfur cycle in coastal marine sediment beneath areas of intensive shellfish aquaculture. Appl Environ Microbiol 71: 2925-2933.

50. Madrid VM, Taylor GT, Scranton MI, Chistoserdov AY (2001) Phylogenetic diversity of bacterial and archaeal communities in the anoxic zone of the Cariaco Basin. Appl Environ Microbiol 67: 1663-1674.

51. Bissett A, Bowman J, Burke C (2006) Bacterial diversity in organically-enriched fish farm sediments. FEMS Microbiol Ecol 55: 48-56.

52. Teske A, Hinrichs KU, Edgcomb V, de Vera Gomez A, Kysela D, et al. (2002) Microbial diversity of hydrothermal sediments in the Guaymas Basin: evidence for anaerobic methanotrophic communities. Appl Environ Microbiol 68: 1994-2007.

53. Hugenholtz P, Pitulle C, Hershberger KL, Pace NR (1998) Novel division level bacterial diversity in a Yellowstone hot spring. J Bacteriol 180: 366-376.

54. Harris JK, Kelley ST, Pace NR (2004) New perspective on uncultured bacterial phylogenetic division OP11. Appl Environ Microbiol 70: 845-849.

55. Chen CL, Wu JH, Tseng IC, Liang TM, Liu WT (2009) Characterization of active microbes in a full-scale anaerobic fluidized bed reactor treating phenolic wastewater. Microbes Environ 24: 144-153.

56. Lau MC, Aitchison JC, Pointing SB (2009) Bacterial community composition in thermophilic microbial mats from five hot springs in central Tibet. Extremophiles 13: 139-149.

57. Sekiguchi Y, Kamagata Y, Syutsubo K, Ohashi A, Harada H, et al. (1998) Phylogenetic diversity of mesophilic and thermophilic granular sludges determined by 16S rRNA gene analysis. Microbiology 144 ( Pt 9): 2655-2665.

58. Wu J, Liu W, Tseng I, Cheng S (2001) Characterization of a 4-methylbenzoate-degrading methanogenic consortium as determined by small-subunit rDNA sequence analysis. J Biosci Bioeng 91: 449-455.

59. Sunna A, Bergquist PL (2003) A gene encoding a novel extremely thermostable 1,4-beta-xylanase isolated directly from an environmental DNA sample. Extremophiles 7: 63-70.

60. Nercessian O, Noyes E, Kalyuzhnaya MG, Lidstrom ME, Chistoserdova L (2005) Bacterial populations active in metabolism of C1 compounds in the sediment of Lake Washington, a freshwater lake. Appl Environ Microbiol 71: 6885-6899.

61. Lymperopoulou DS, Kormas KA, Karagouni AD (2012) Variability of prokaryotic community structure in a drinking water reservoir (Marathonas, Greece). Microbes Environ 27: 1-8.

62. Omoregie EO, Mastalerz V, de Lange G, Straub KL, Kappler A, et al. (2008) Biogeochemistry and community composition of iron- and sulfur-precipitating microbial mats at the Chefren mud volcano (Nile Deep Sea Fan, Eastern Mediterranean). Appl Environ Microbiol 74: 3198-3215.

63. Ye W, Liu X, Lin S, Tan J, Pan J, et al. (2009) The vertical distribution of bacterial and archaeal communities in the water and sediment of Lake Taihu. FEMS Microbiol Ecol 70: 107-120.

64. Lopez-Garcia P, Gaill F, Moreira D (2002) Wide bacterial diversity associated with tubes of the vent worm Riftia pachyptila. Environ Microbiol 4: 204-215.

65. Dhillon A, Teske A, Dillon J, Stahl DA, Sogin ML (2003) Molecular characterization of sulfate-reducing bacteria in the Guaymas Basin. Appl Environ Microbiol 69: 2765-2772.

66. Chouari R, Le Paslier D, Daegelen P, Ginestet P, Weissenbach J, et al. (2005) Novel predominant archaeal and bacterial groups revealed by molecular analysis of an anaerobic sludge digester. Environ Microbiol 7: 1104-1115.

67. Riviere D, Desvignes V, Pelletier E, Chaussonnerie S, Guermazi S, et al. (2009) Towards the definition of a core of microorganisms involved in anaerobic digestion of sludge. ISME J 3: 700-714.
